# Supplementary figures and images for: Penalised regression improves imputation of cell-type specific expression using RNA-seq data from mixed cell populations compared to domain-specific methods
Source: PLoS Comput Biol. 2025 Mar 7;21(3):e1012859. doi: 10.1371/journal.pcbi.1012859 (PMC11957391; doi:10.1371/journal.pcbi.1012859)

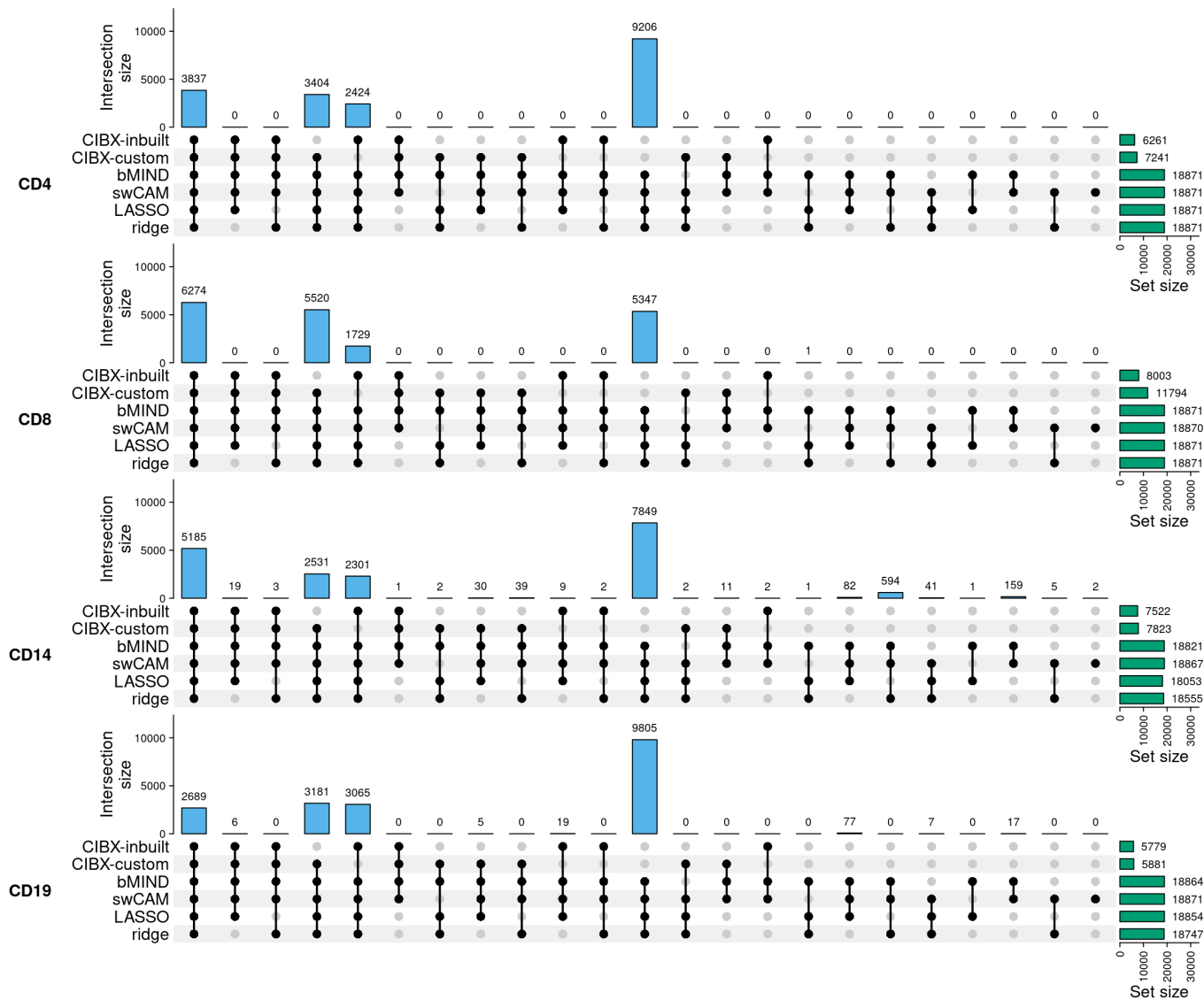

Supplement: S1 Fig — Predicted genes were defined as those with variations in expression across subjects. For each panel (cell type), the right bar plot indicates the numbers of predicted genes (No.Pred.Genes) by approach, and the top bar plot demonstrates No.Pred.Genes common in different combinations of approaches (black dots), but not in the grey-dot approaches, if present. (PDF) [file pcbi.1012859.s001.pdf]

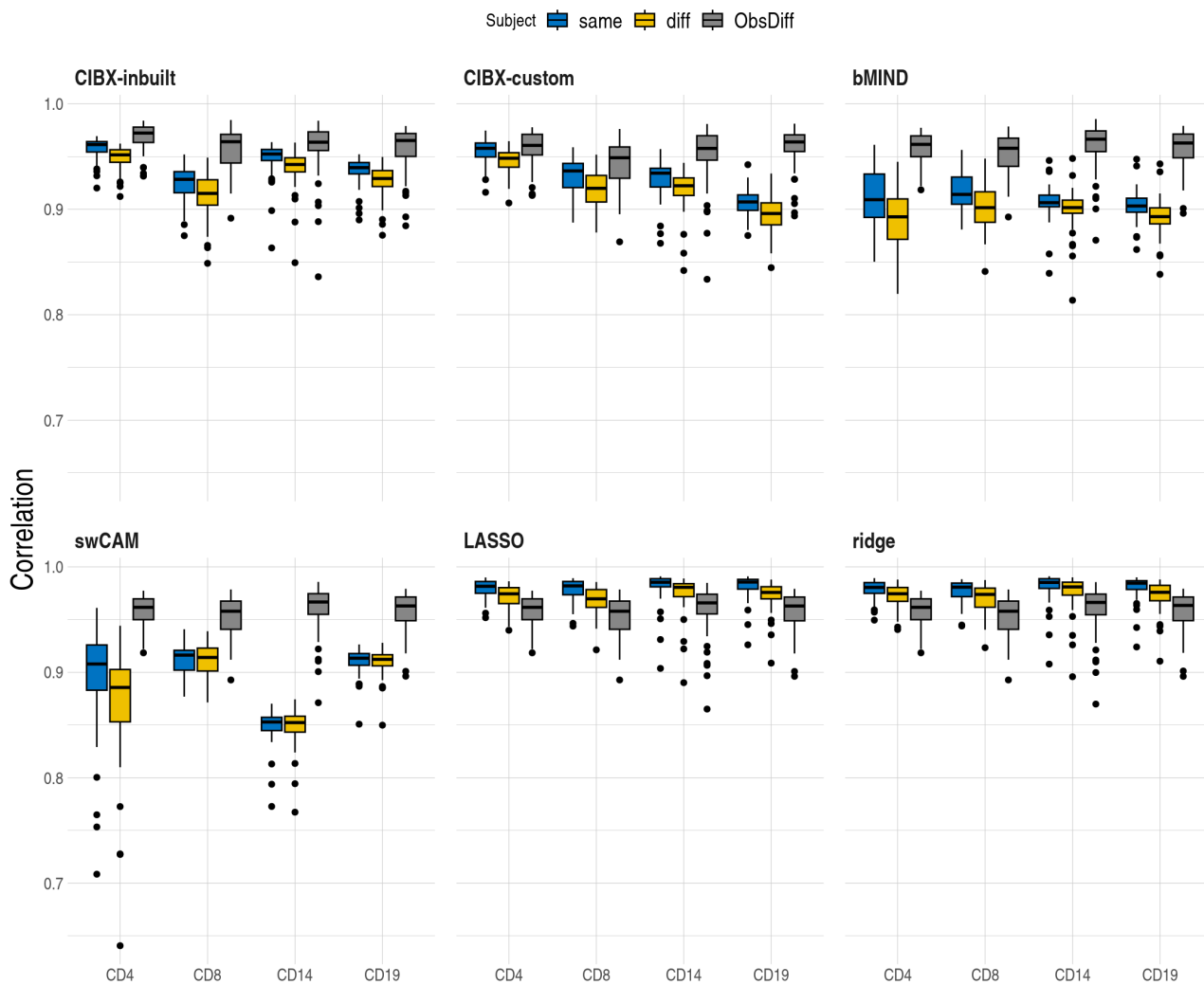

Supplement: S2 Fig — One estimate per subject. CIBX-inbuilt: CIBERSORTx with the inbuilt signature matrix; CIBX-custom: CIBERSORTx with a custom signature matrix derived from sorted cell-type expression in training samples; bMIND: bMIND with flow fractions; swCAM: swCAM with flow fractions; LASSO/ridge: regularised multi-response Gaussian models. (PDF) [file pcbi.1012859.s002.pdf]

A

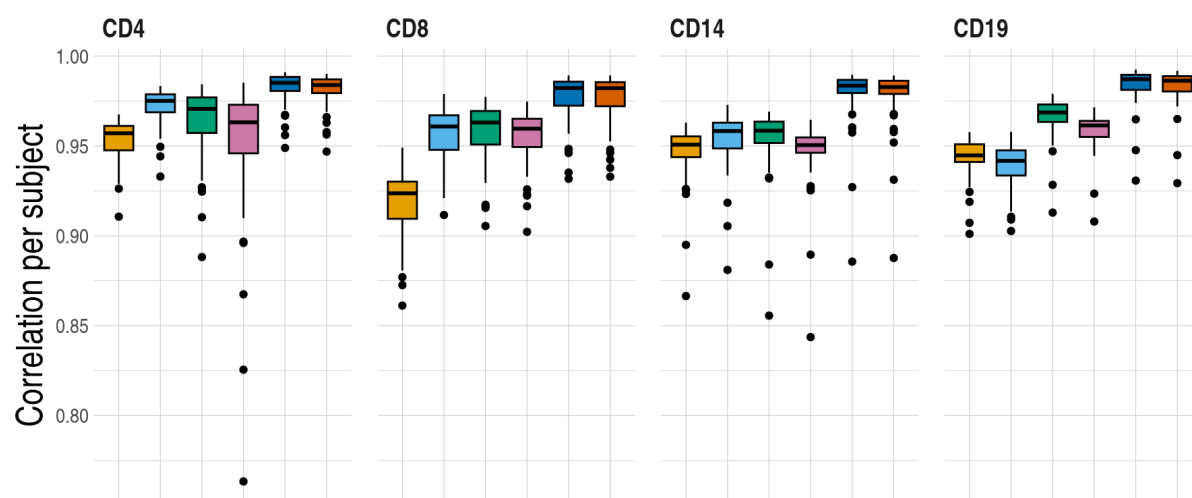

B

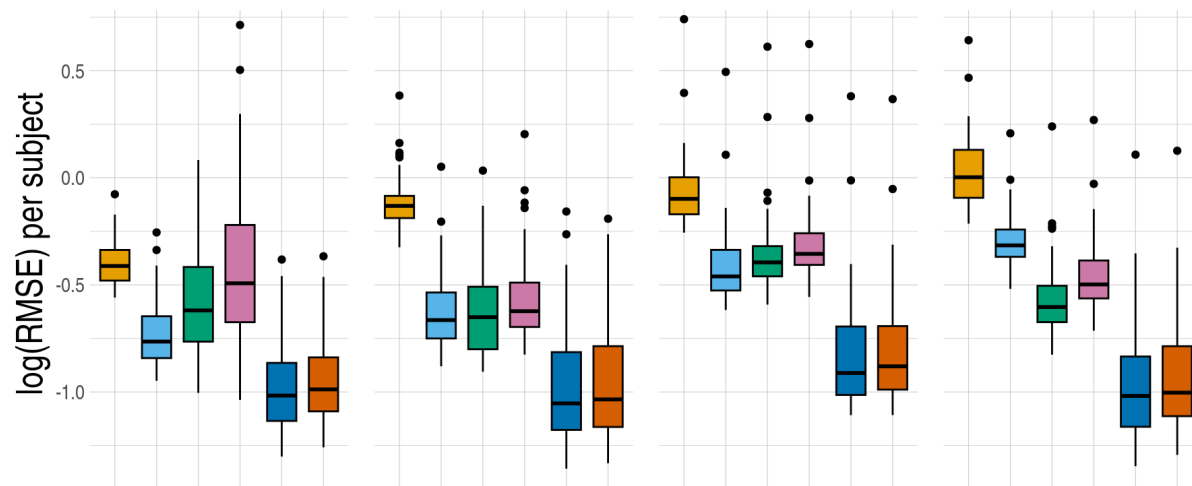

C

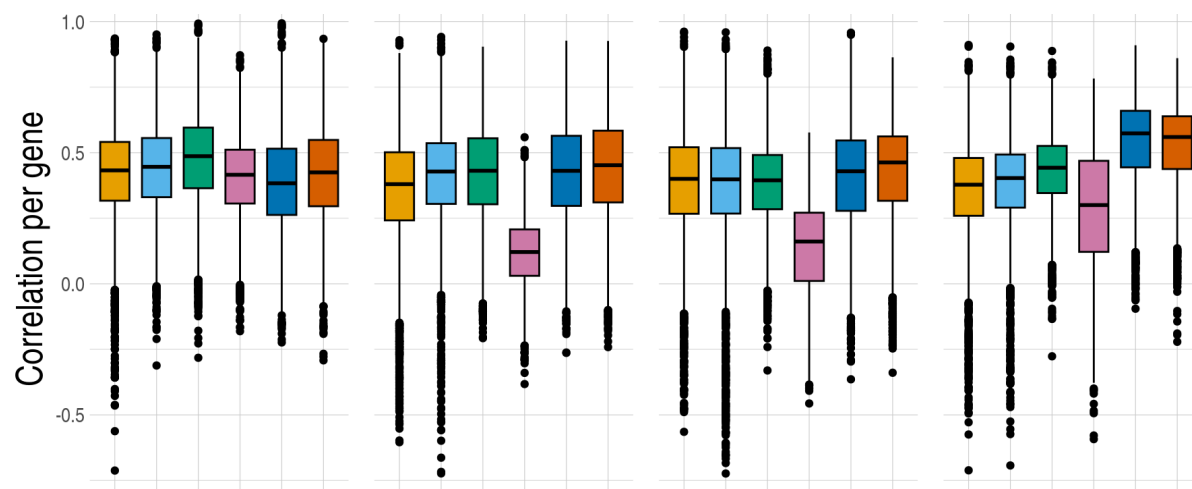

D

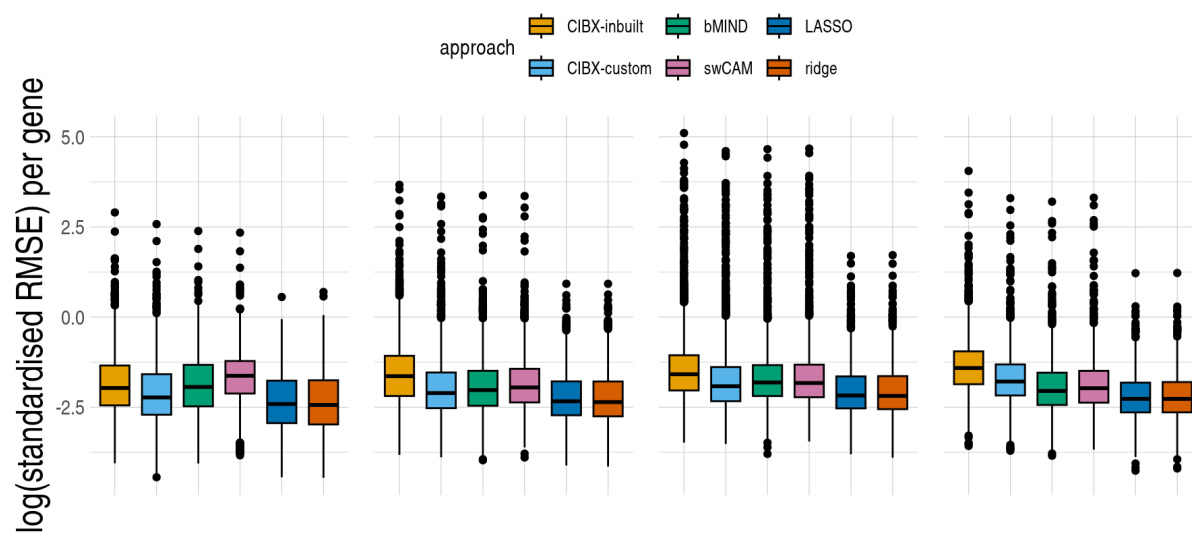

Supplement: S3 Fig — For each cell type, genes common across approaches were used. No. common genes are 3837 for CD4, 6274 for CD8, 5185 for CD14, and 2689 for CD19, respectively. (A) Pearson correlation and (B) log root mean square error (RMSE) comparing observed to predicted cell-type expression of genes from the same subjects, one estimate per subject. (C) Pearson correlation and (D) log RMSE between observed and predicted cell-type expression across testing samples for each gene, estimate per gene. RMSE was standardised by the average observed expression per gene. CIBX-inbuilt: CIBERSORTx expression deconvolution with the inbuilt signature matrix; CIBX-custom: CIBERSORTx expression deconvolution with a custom signature matrix derived from sorted cell-type expression in training samples; bMIND: bMIND expression deconvolution with flow fractions; swCAM: swCAM deconvolution with flow fractions; LASSO/ridge: expression predicted from regularised multi-response Gaussian models. (PDF) [file pcbi.1012859.s003.pdf]

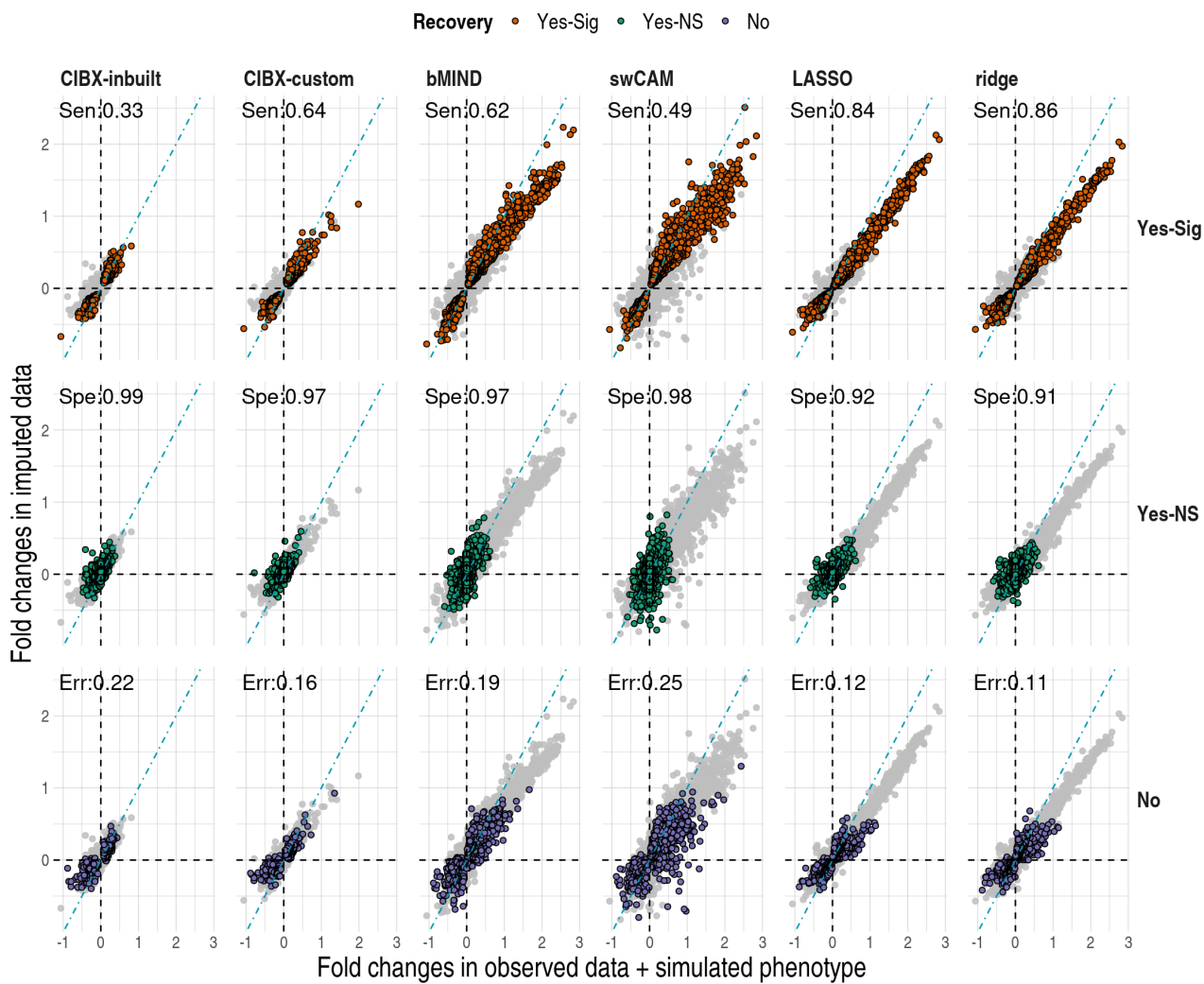

Supplement: S4 Fig — DGE analysis was carried out using limma based on one of the simulated phenotypes. An FDR of 0.05 was used in both observed and imputed data here, and CD4 DGE recovery is shown. DGE results in each method (column) are the same, with coloured points for genes falling into that category of recovery status (row) and grey points for genes not belonging to the same category. Recovery status: No, Yes-NS, and Yes-Sig. Yes-Sig (sensitivity; Sen): differentially expressed genes in the observed data were also called significant in the imputed data, and the orientations of the effect sizes are the same in both data. Yes-NS (specificity; Spe): genes are called non-significant (NS) in both data. No (error; Err): misclassified genes; Err is calculated as the percentage of misclassified genes to the total number of predicted genes. (PDF) [file pcbi.1012859.s004.pdf]

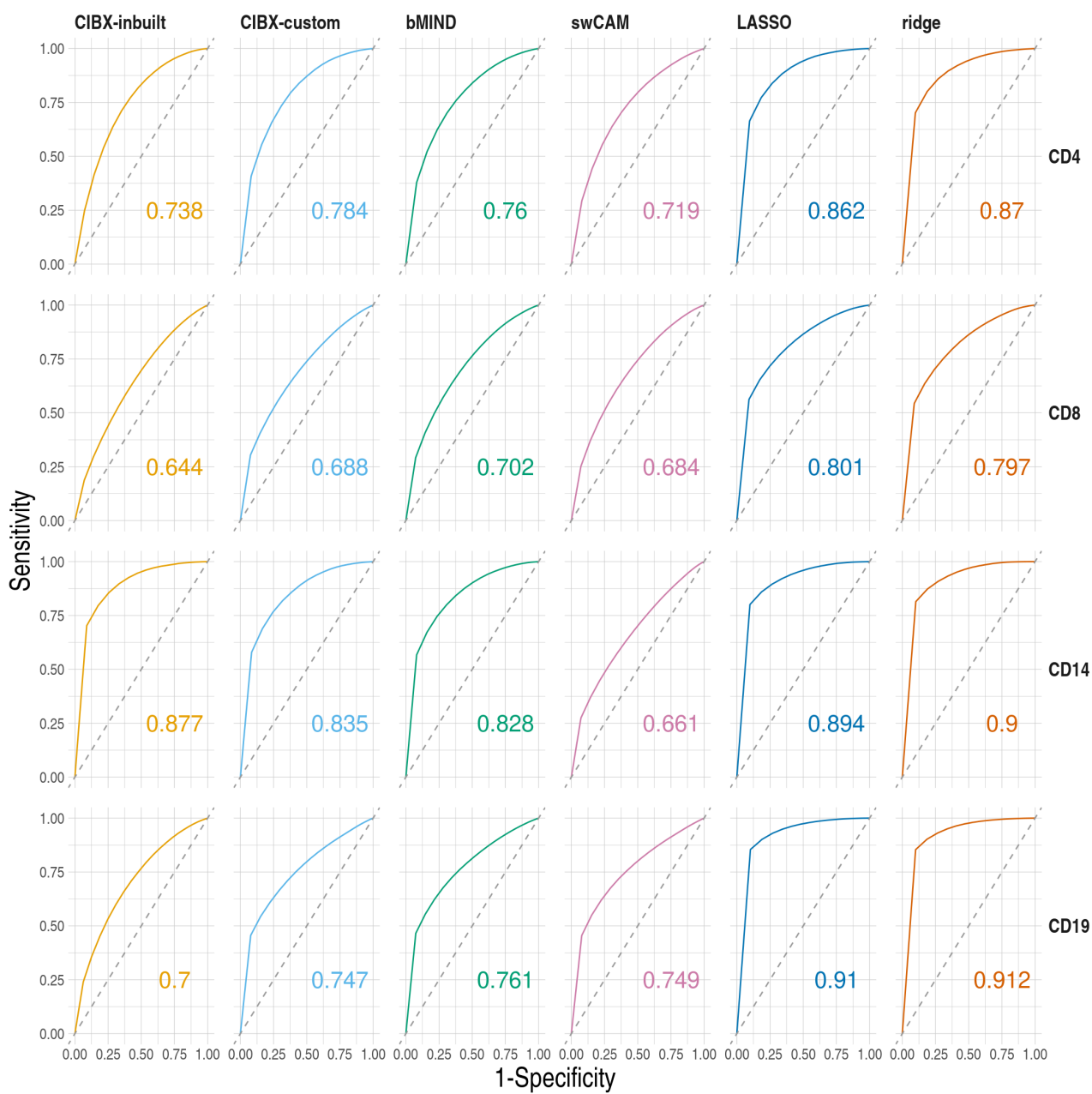

Supplement: S5 Fig — FDR was fixed at 0.05 in the observed data and varied from 0 to 1 by 0.05 in the imputed data. Dashed lines indicate y = x. (PDF) [file pcbi.1012859.s005.pdf]

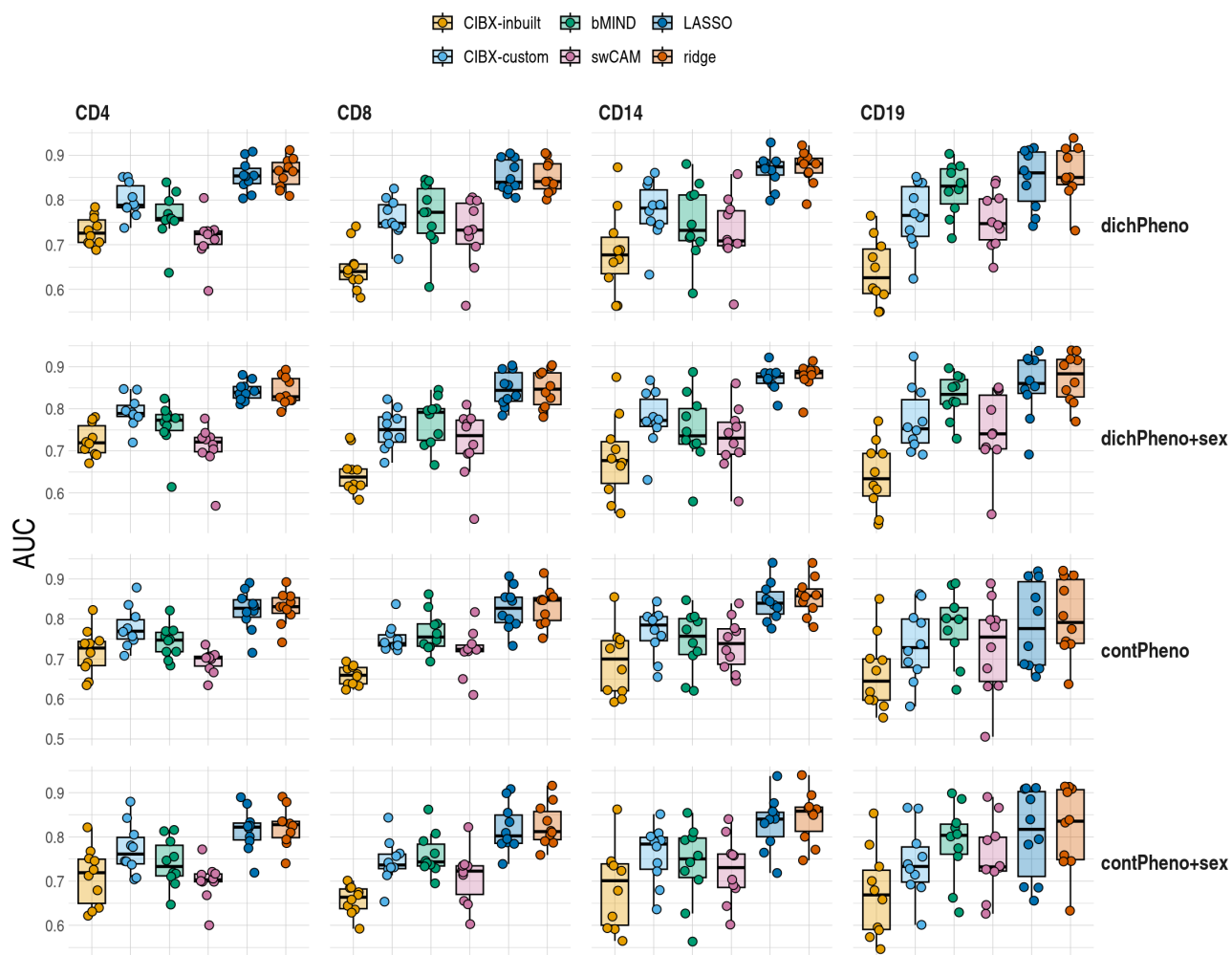

Supplement: S6 Fig — Area under curve (AUC) distributions by approach and cell type (columns) for each scenario (rows). Scenarios differed in simulated dichotomous (dichPheno)/ continuous (contPheno) phenotypes, with/without sex as a covariate in the DGE analysis of the common gene sets across approaches. No. common genes are 3837 for CD4, 6274 for CD8, 5185 for CD14, and 2689 for CD19. dichPheno: dichotomous phenotype; dichPheno+sex: simulated dichotomous phenotype and sex; contPheno: continuous phenotype; contPheno+sex: continuous phenotype and sex. Each point is a simulated phenotype, and there are ten simulated phenotypes. For each simulated phenotype, the receiver operating characteristic curve and AUC were estimated by FDR fixed at 0.05 in the observed data and varied FDRs from 0 to 1 by 0.05 in the imputed data. Box plots showed the AUC distributions, with horizontal lines from the bottom to the top for 25%, 50% and 75% quantiles, respectively. CIBX-inbuilt: CIBERSORTx with the inbuilt signature matrix; CIBX-custom: CIBERSORTx with a custom signature matrix; bMIND: bMIND with flow fractions; swCAM: swCAM with flow fractions; LASSO/ridge: regularised multi-response Gaussian models. (PDF) [file pcbi.1012859.s006.pdf]

A

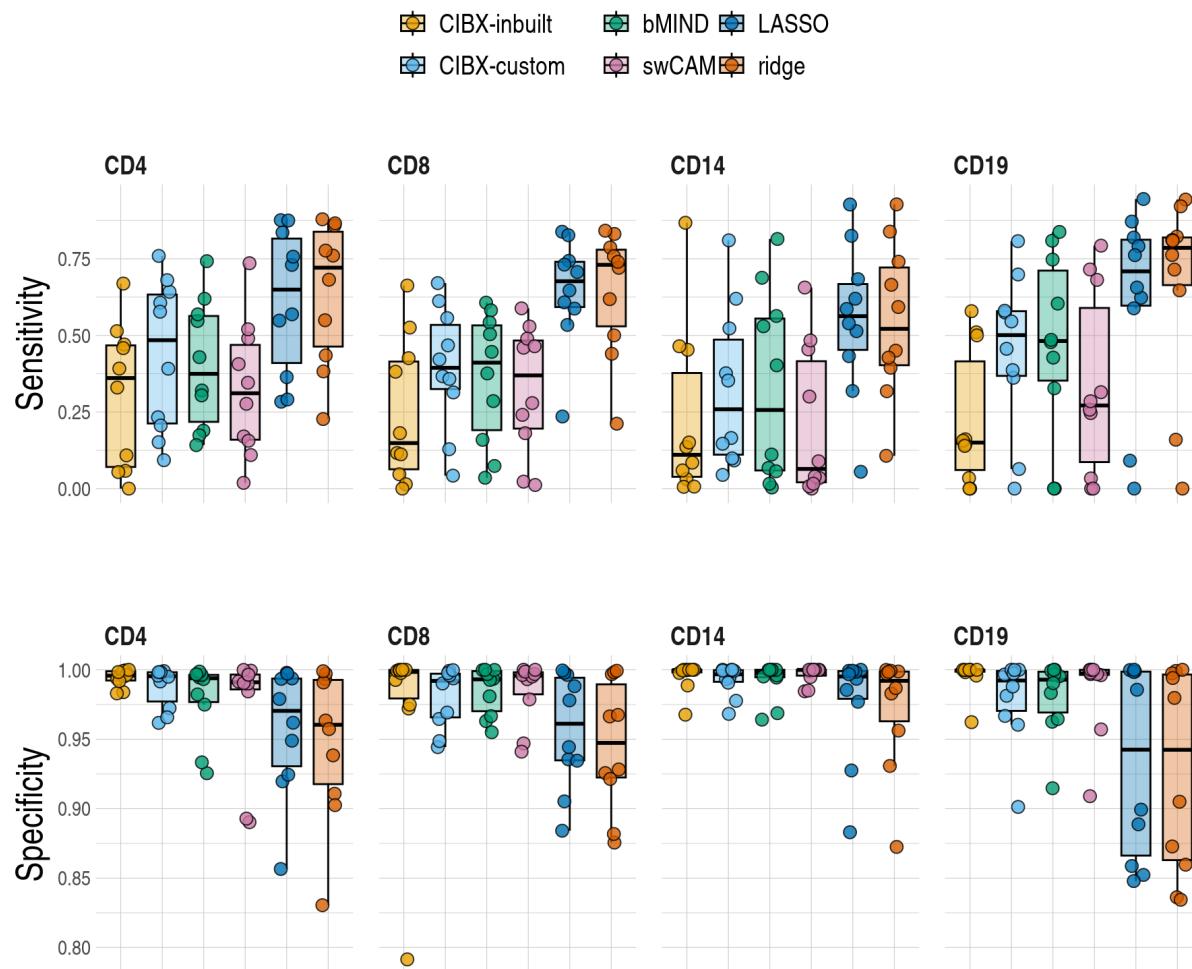

B

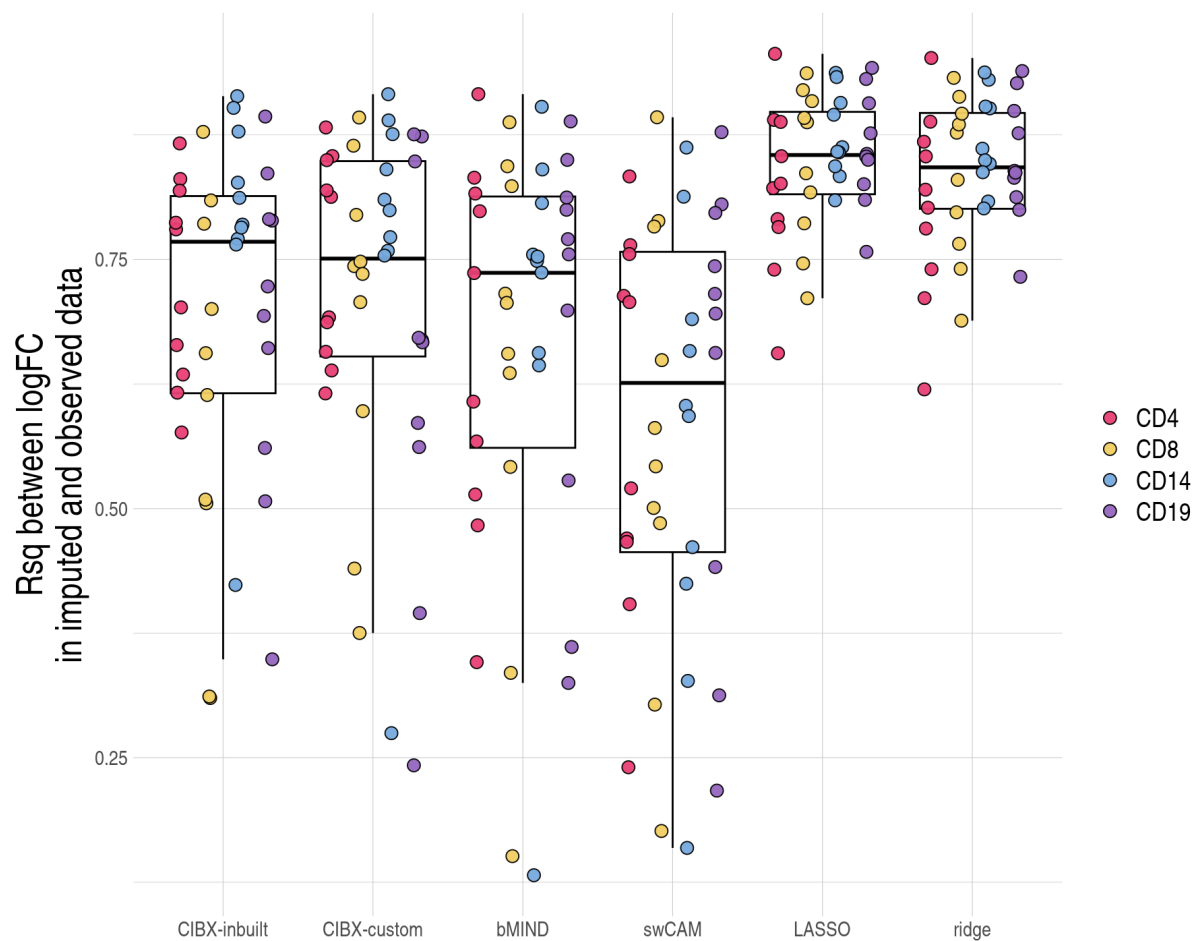

C

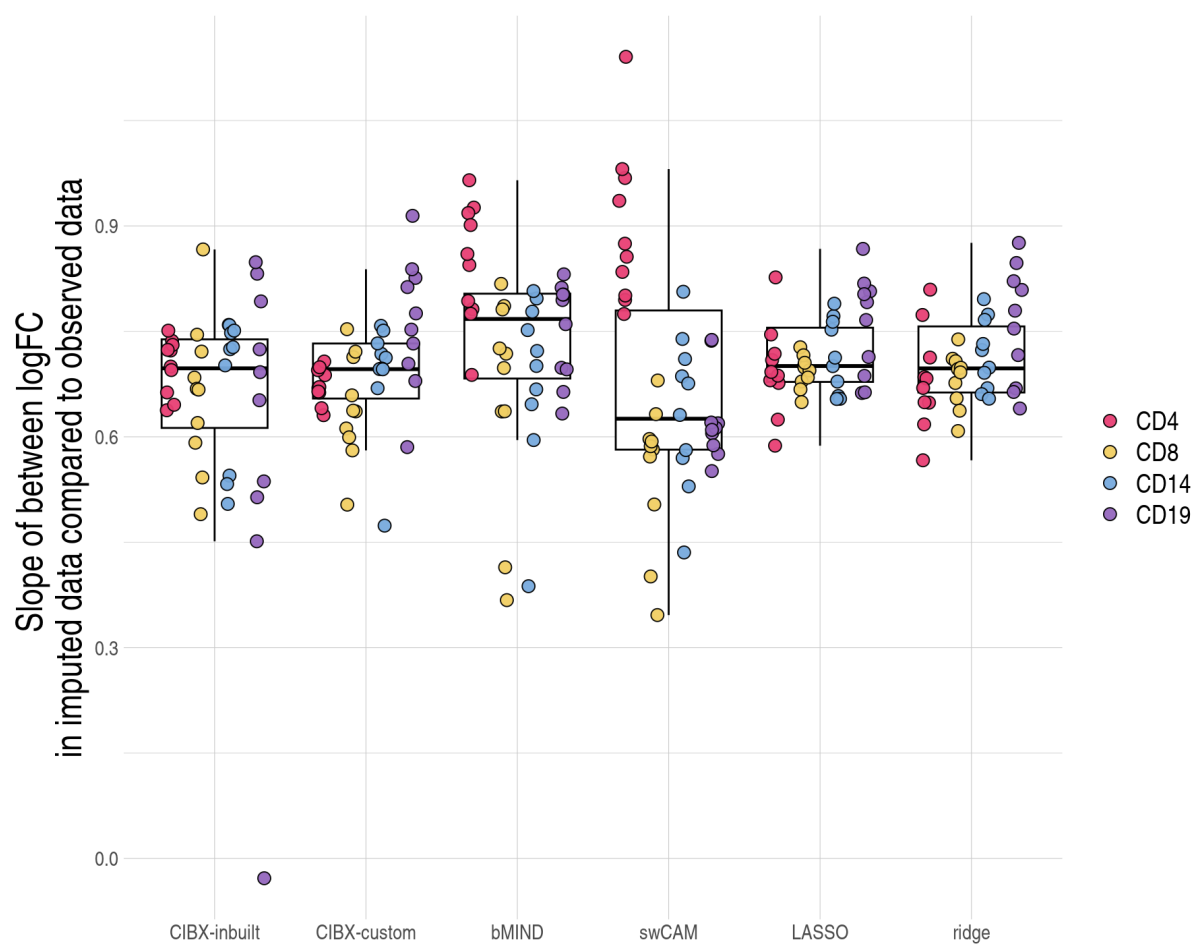

Supplement: S7 Fig — (A) Distributions of sensitivity and specificity of DGE recovery by cell type and approach. Each point is a simulated phenotype. (B) R-squared (Rsq, y-axis) and (C) slopes of imputed log2 fold changes (FC) regression on observed effect sizes by approach. Each point is a simulated phenotype, coloured by cell type. CIBX-inbuilt: CIBERSORTx with the inbuilt signature matrix; CIBX-custom: CIBERSORTx with a custom signature matrix derived; bMIND: bMIND with flow fractions; swCAM: swCAM with flow fractions; LASSO/ridge: regularised multi-response Gaussian models. (PDF) [file pcbi.1012859.s007.pdf]

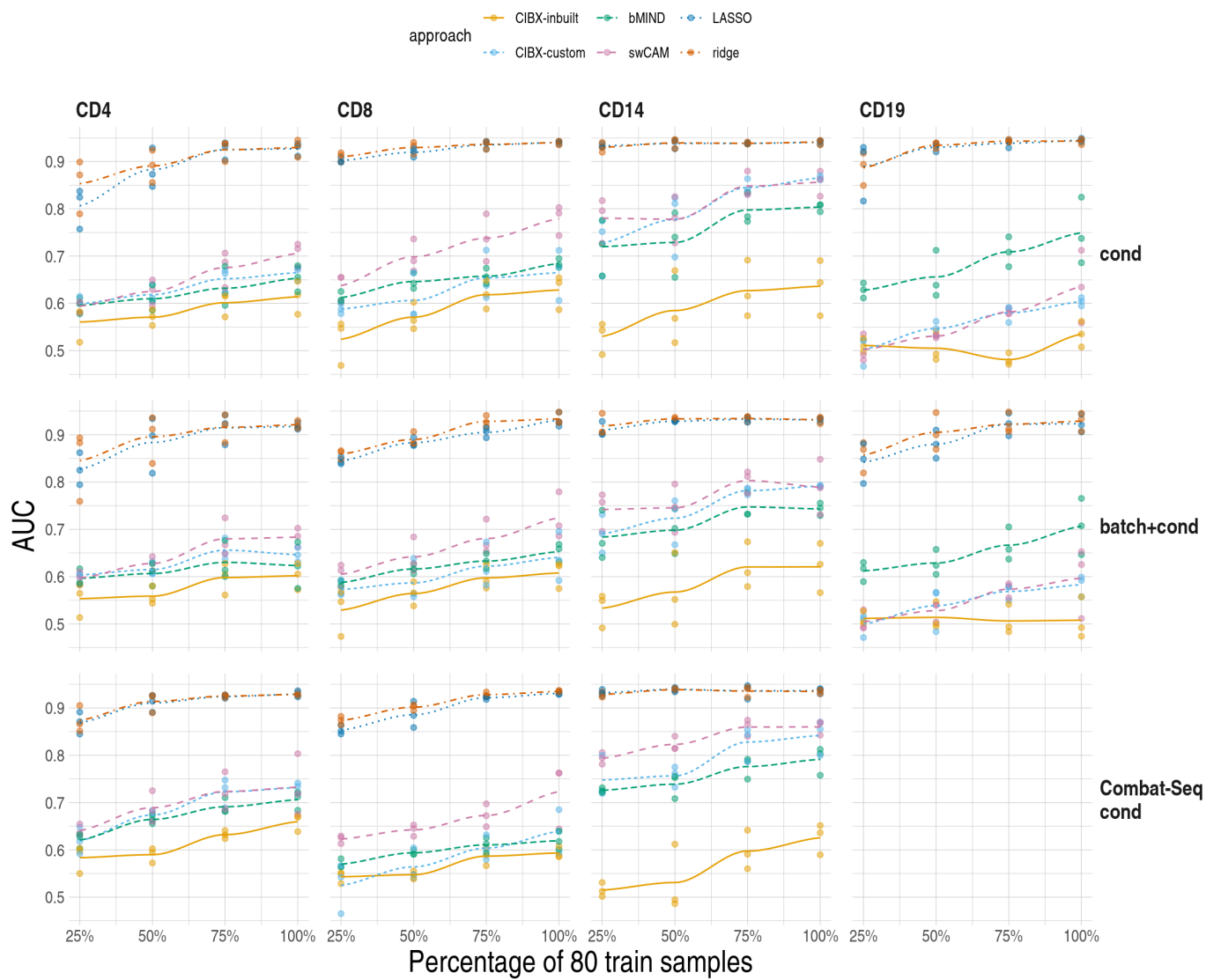

Supplement: S8 Fig — We varied training sample size from 20 (25%) to 80 (100%) (x-axis) and quantified area under curve (AUC) for DGE recovery (y-axis) by cell type (columns) for each scenario (rows). cond: raw aggregated read counts were used for DGE analysis of in vitro stimulation with C. albicans after 3 hours (3hCA) vs untreated (UT). batch+cond: same as cond, and with batch (V2 & V3 chemistry) as a covariate in the 3hCA vs UT DGE model. Combat-seq cond: Combat-seq batch-corrected read counts were used in DGE analysis of 3hCA vs UT. DGE analysis was performed in the training and testing samples and restricted to common gene sets predicted by all methods. Each point is the result of one analysis, and three replicates were conducted for each training sample size. For each pseudobulk data, the receiver operating characteristic curve and AUC were estimated by FDR fixed at 0.05 in the observed DGE results and varied FDRs from 0 to 1 by 0.05 in the DGE results using imputed expression. Local polynomial regression fitting (loess) were plotted for each approach. Noted that no data is shown for CD19/Combat-Seq cond because the common gene set predicted by all methods equals zero. CIBX-inbuilt: CIBERSORTx with the inbuilt signature matrix; CIBX-custom: CIBERSORTx with a custom signature matrix based on pure cell expression in the training samples; bMIND: bMIND with true fractions; swCAM: swCAM with true fractions; LASSO/ridge: regularised multi-response Gaussian models. (PDF) [file pcbi.1012859.s008.pdf]

approach

- CIBX-inbuilt
- bMIND
- LASSO
- CIBX-custom
- swCAM
- ridge

**A**

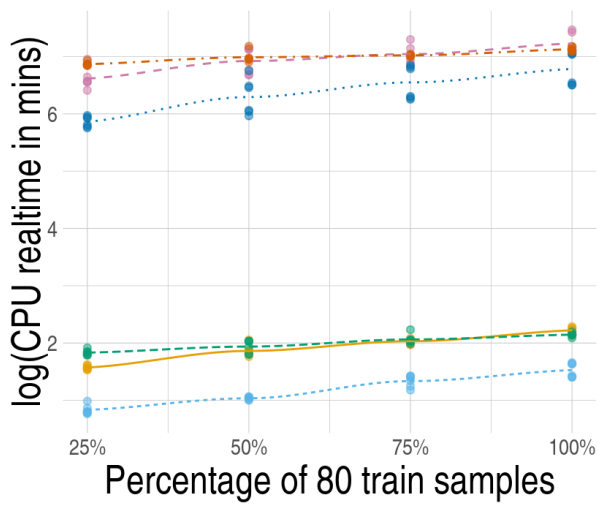

**B**

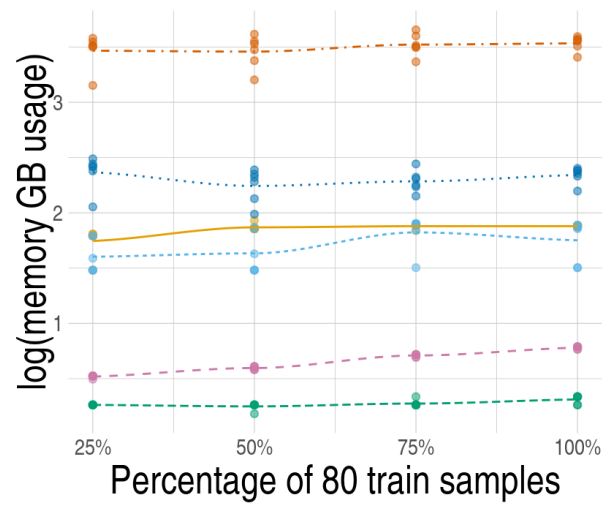

Supplement: S9 Fig — (A) CPU time in minutes (B) memory GB usage in the nature logarithm scale (y-axis) across 25%, 50%, 75% and 100% percentage of 80 train samples (x-axis). For serial jobs, swCAM, LASSO and ridge, CPU time was summed together for the same pseudobulk data, and median of memory usage was taken per pseudobulk data. Each point is a pseudobulk data. Local polynomial regression fitting (loess) were plotted for each approach. CIBX-inbuilt: CIBERSORTx with the inbuilt signature matrix; CIBX-custom: CIBERSORTx with a custom signature matrix generated using pure cell expression of the training samples in the pseudobulk data; bMIND: bMIND with true fractions; swCAM: swCAM with true fractions; LASSO/ridge: regularised multi-response Gaussian models. (PDF) [file pcbi.1012859.s009.pdf]

A

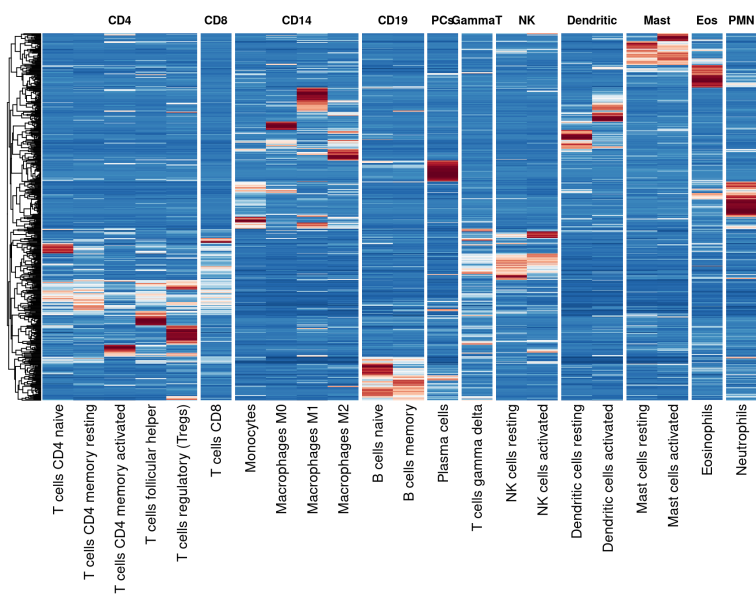

B

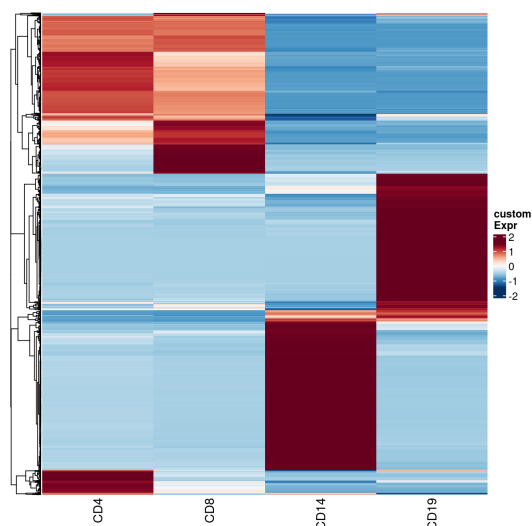

C

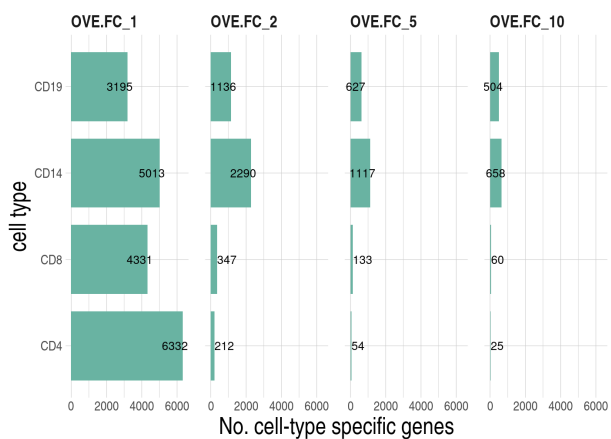

D

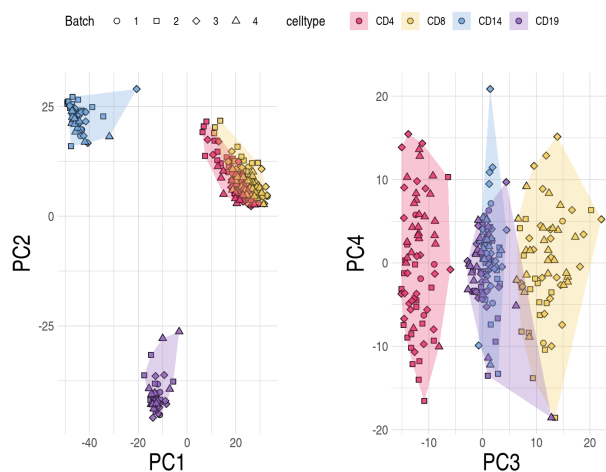

E

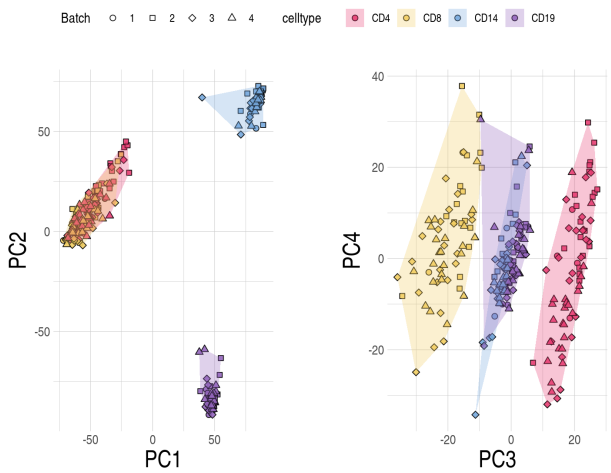

F

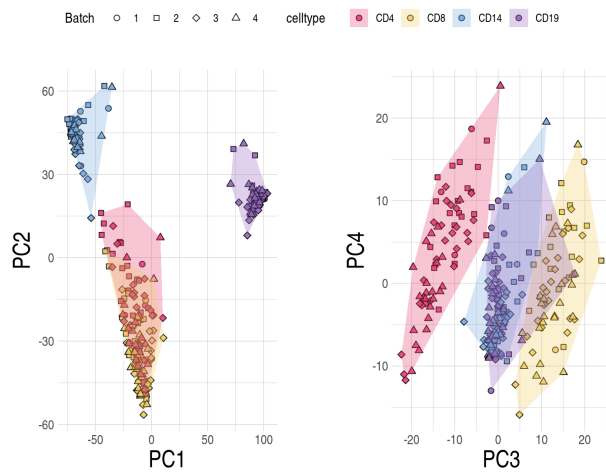

Supplement: S10 Fig — (A) Expression of inbuilt signature genes (N=547) in 22 leukocyte subsets, curated by the CIBERSORTx team from microarray gene expression. For each gene (row), expression is centred to the mean and scaled by the standard deviation across cell types (column). Columns are split by the collapsed classes. CD4: T cells CD4 naive, T cells CD4 memory resting, T cells CD4 memory activated, T cells follicular helper, and T cells regulatory (Tregs); CD8: T cells CD8; CD14: Monocytes, Macrophages M0, Macrophages M1, and Macrophages M2; CD19: B cells naive and B cells memory; PCs: Plasma cells; GammaT: T cells gamma delta; NK: NK cells resting and NK cells activated; Dendritic: Dendritic cells resting and Dendritic cells activated; Mast: Mast cells resting and Mast cells activated; Eos: EosinophilsPMN: Neutrophils (B) Expression of custom signature genes (N=1589), derived from our sorted-cell RNAseq expression in 80 training subjects using CIBERSORTx. Expression is centred and scaled across cell types (column) by gene (row). (C) Numbers (No.) of debCAM cell-type specific genes by cell type (row) and selection criteria (column). debCAM, which does not have the signature matrix, selects the cell-type-specific genes, that are over-expressed in one cell type versus everyone (OVE). OVE fold change (FC) of 1, 2, 5 and 10 were used in our sorted cell expression of 80 training subjects. The first four principal components from PCA analysis of (D) inbuilt, (E) custom signature and (F) debCAM cell type specific gene expression in test samples. Each dot is a sample, coloured by cell type and shaped by sequencing batch. (PDF) [file pcbi.1012859.s010.pdf]

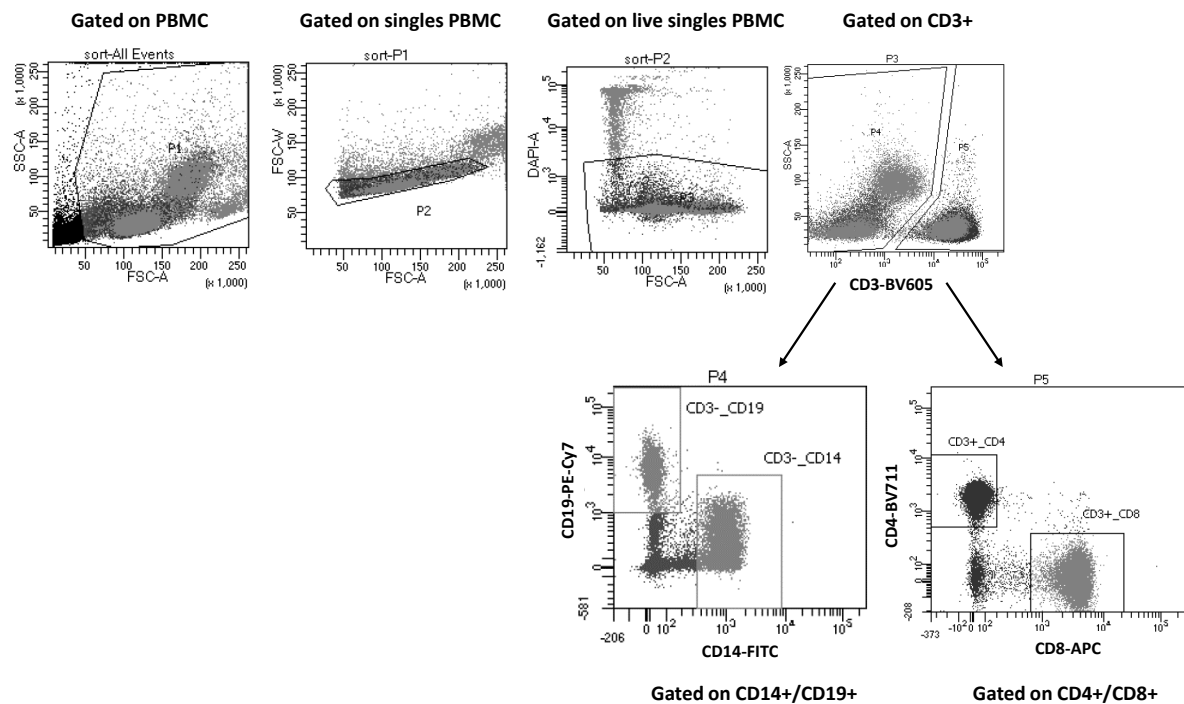

Supplement: S11 Fig — Initial gating was performed with forward (FSC) and side (SSC) scatters to isolate lymphocytes (PBMC). Further gating with FSC-A and FSC-W was done to exclude doublets. Live cells were selected based on the gating with DAPI. The live cells were gated on CD3 to separate between CD3+ and CD3- cells. The CD3+ population was further gated for CD4 and CD8, whilst the CD3- population was gated for CD14 and CD19. (PDF) [file pcbi.1012859.s011.pdf]

A

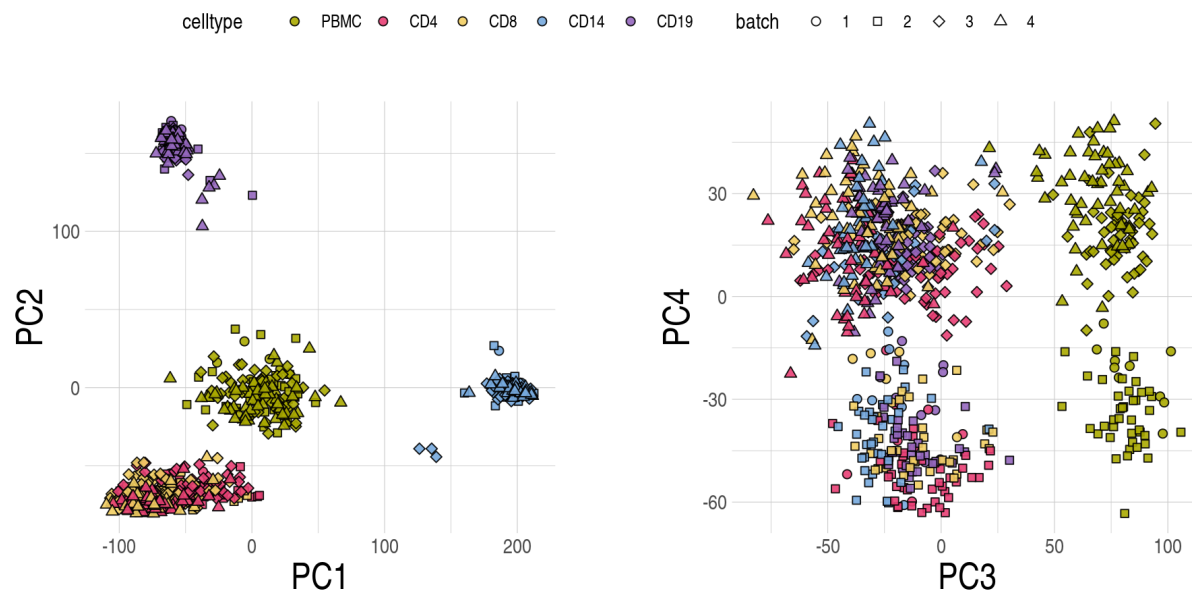

B

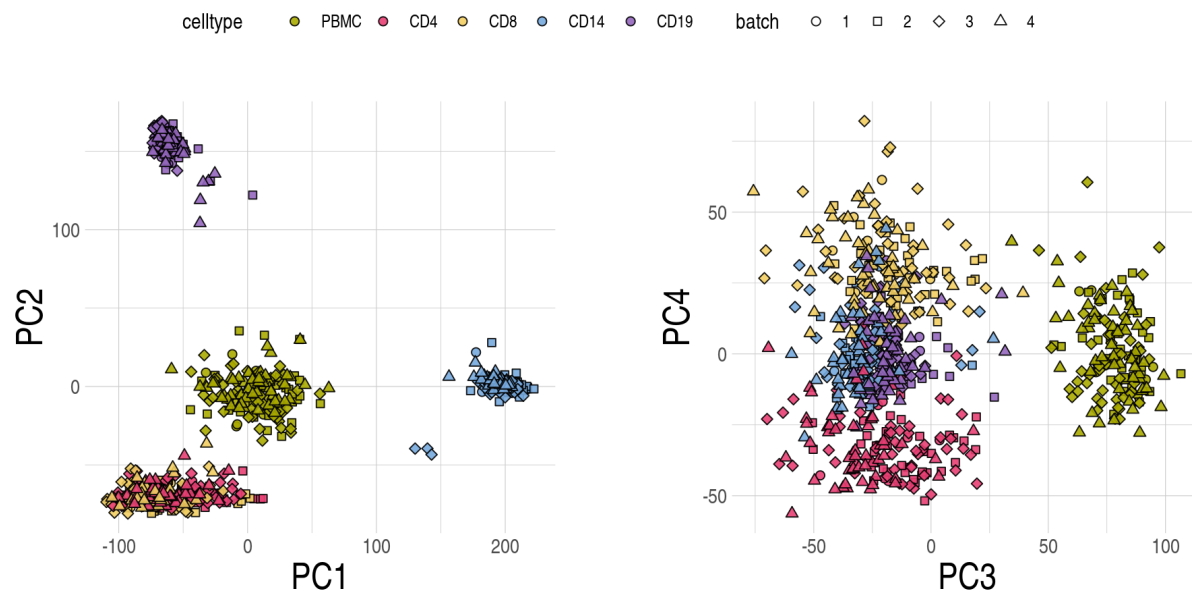

Supplement: S12 Fig — (PDF) [file pcbi.1012859.s012.pdf]

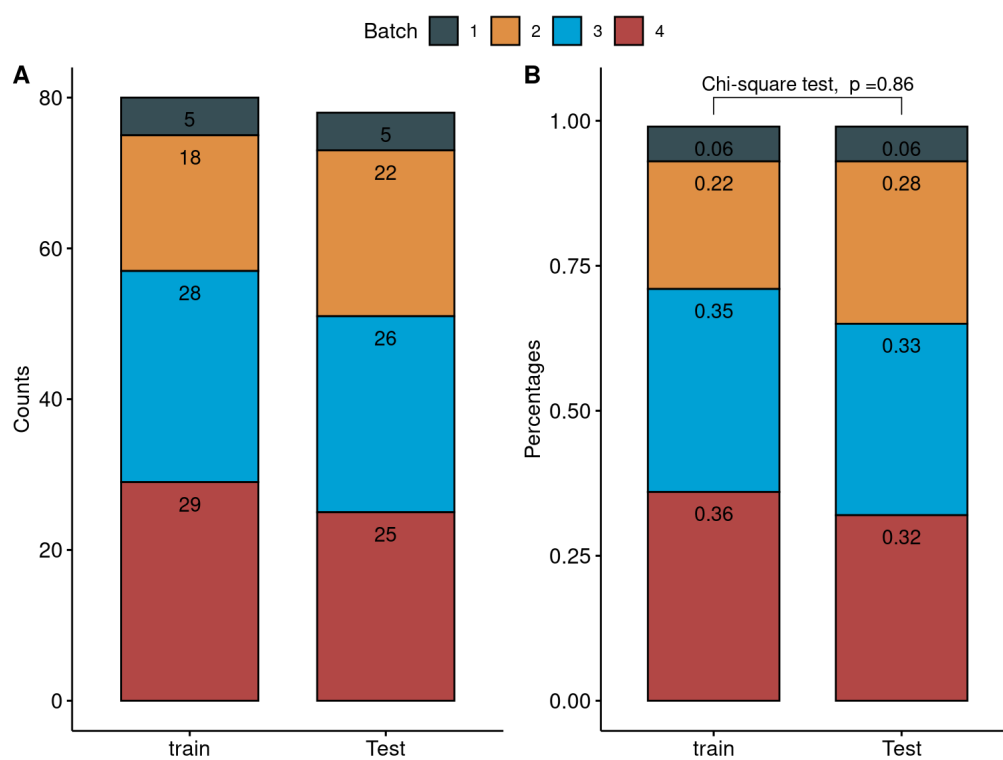

Supplement: S13 Fig — (PDF) [file pcbi.1012859.s013.pdf]

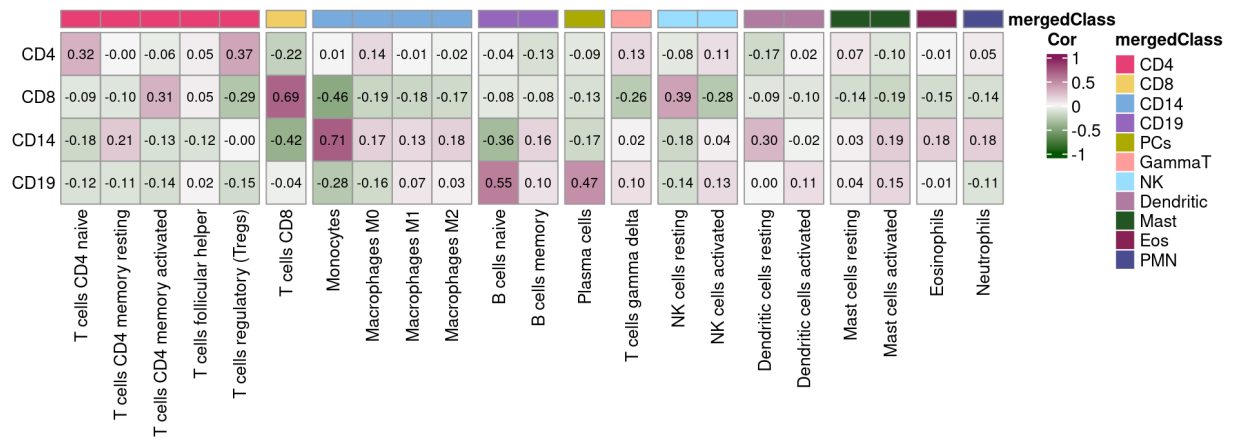

Supplement: S14 Fig — Columns are LM22 cell subsets split by their merged classes (top annotation). Rows are ground-truth cell types. Each cell is coloured based on the strength of the correlation. (PDF) [file pcbi.1012859.s014.pdf]
