# Supplementary material for: Penalised regression improves imputation of cell-type specific expression using RNA-seq data from mixed cell populations compared to domain-specific methods
Source: PLoS Comput Biol. 2025 Mar 7;21(3):e1012859. doi: 10.1371/journal.pcbi.1012859 (PMC11957391; doi:10.1371/journal.pcbi.1012859)
Supplement: S1 Table — (PDF) [file pcbi.1012859.s015.pdf]

S1 Table. Summary of existing deconvolution approaches

Approach/matrices	signature genes *H*	cell fractions *F*	average cell-type expression *H1*	sample-level cell-type expression *G*	evaluation in this study
CIBERSORTx	DGE[a]	ν vector regression	NNLS	NNLS	Yes
bMIND	x	NNLS	BLMM	BLMM	Yes
debCAM/swCAM	OVE.FC[b]	pcaPP::l1median[b]	NNLS	LMM	Yes
MIND	x	NNLS	EB-GLM[c]	EB-GLM[c]	No
CellR	x	LP[d]	x	SA[d]	No
Rodeo	x	x	NNLS	x	No
csSAM	x	x	NNLS	x	No
EPIC	x	NNLS	x	x	No
quanTIseq	x	NNLS	x	x	No
FARDEEP	x	NNLS	x	x	No

NNLS: non-negative least squares, noted that NNLS implementation differ by approach; BLMM: Bayesian mixed-effects model

[a] differential gene expression (DGE) analysis of purified cell populations

[b] Genes with fold changes (FC) over the predefined threshold in the comparisons of one verse everyone (OVE) cell populations; pcaPP::l1median: spatial median projection; LMM: linear mixed model

[c] EB-GLM: empirical Bayes generalised linear model and it requires multiple RNA-seq expression measurements per subjects

[d] LP: linear programming; simulated annealing (SA) algorithm and it requires predefined cell-type clusters in scRNA-seq data
