# Supplementary material for: Penalised regression improves imputation of cell-type specific expression using RNA-seq data from mixed cell populations compared to domain-specific methods
Source: PLoS Comput Biol. 2025 Mar 7;21(3):e1012859. doi: 10.1371/journal.pcbi.1012859 (PMC11957391; doi:10.1371/journal.pcbi.1012859)
Supplement: S2 Table — (PDF) [file pcbi.1012859.s016.pdf]

S2 Table. Computational time and memory usage by approach based on the CLUSTER data

approach	No. CPUs	CPU time (minutes)	memory usage (Gb)	No. chunks[b]	CPU time chunks[c] Q50 (Q25, Q75)
CIBX-inbuilt	8	8.80	11.0	-	-
CIBX-custom	8	4.45	5.5	-	-
bMIND	4	12.00	1.8	-	-
swCAM	10/1[a]	1713.13[b]	2.95[b]	-	-
LASSO	1	624.22[b]	16.7[b]	304	1.84 (1.23,2.73)
ridge	1	2928.45[b]	58.5[b]	304	8.85 (6.09,12.78)

No.CPUs: CPUs assigned; memory usage: physical RAM memory and swap disk space. Q50 (Q25, Q75): median (25%,75% quantiles); - : not applicable

[a] 10 CPUs for cross-validation and 1 CPU for predicting sample-level cell type expression

[b] Serial jobs: CPU time was summed together, and the median memory usage between jobs was displayed

[c] LASSO and ridge only
