# Supplementary material for: Penalised regression improves imputation of cell-type specific expression using RNA-seq data from mixed cell populations compared to domain-specific methods
Source: PLoS Comput Biol. 2025 Mar 7;21(3):e1012859. doi: 10.1371/journal.pcbi.1012859 (PMC11957391; doi:10.1371/journal.pcbi.1012859)
Supplement: S3 Table — (PDF) [file pcbi.1012859.s017.pdf]

S3 Table. Computational time and memory usage by approach based on the eQTLgen pseudobulk data

approach	No. datasets	No. CPUs	CPU time (minutes)	memory usage (Gb)	No. chunks[c]	CPU time chunks[c] Q50 (Q25, Q75)
CIBX-inbuilt	24	4	7.22 (5.64,8.12)	6.45 (6.25,6.6)	-	-
CIBX-custom	24	4	3.08 (2.71,4.08)	6 (4.47,6.53)	-	-
bMIND	24	4	7.61 (6.33,8.18)	1.3 (1.3,1.3)	-	-
swCAM	24	10/1[a]	1103.68 (799.36,1256.04)[b]	1.92 (1.77,2.07)[b]	-	-
LASSO	24	1	592.13 (391.41,862.2)[b]	10.4 (9.46,10.94)[b]	5377	1.88 (0.54,3.85)
ridge	24	1	1103.6 (1017.01,1199.88)[b]	33.6 (32.83,35.35)[b]	5377	4.62 (2.1,7.25)

No.CPUs: CPUs assigned; memory usage: physical RAM memory and swap disk space; Numbers in CPU time and memory usage indicate median (25%, 75% quantiles) across 24 pseudobulk data

[a] 10 CPUs for cross-validation and 1 CPU for predicting sample-level cell type expression

[b] Serial jobs: CPU time was summed together, and the median memory usage between jobs was taken on a pseudobulk data basis.

[c] LASSO and ridge only; No.chunks: total chunks across 24 pseudobulk data. CPU time was summarised in a chunk-level
