# Supplementary material for: Penalised regression improves imputation of cell-type specific expression using RNA-seq data from mixed cell populations compared to domain-specific methods
Source: PLoS Comput Biol. 2025 Mar 7;21(3):e1012859. doi: 10.1371/journal.pcbi.1012859 (PMC11957391; doi:10.1371/journal.pcbi.1012859)
Supplement: Attachment — Submitted filename: PLOS_comBioRevision.pdf [file pcbi.1012859.s019.pdf]

Dear Wallace,

Thank you very much for submitting your manuscript "Penalised regression improves imputation of cell-type specific expression using RNA-seq data from mixed cell populations compared to domain-specific methods" for consideration at PLOS Computational Biology.

As with all papers reviewed by the journal, your manuscript was reviewed by members of the editorial board and by several independent reviewers. In light of the reviews (below this email), we would like to invite the resubmission of a significantly-revised version that takes into account the reviewers' comments.

As you see, all three reviewers found your work interesting and a good fit to PLOS CB, but they had important comments on the methods and on the writing. I am confident that taking these comments into account will significantly improve your manuscript.

We cannot make any decision about publication until we have seen the revised manuscript and your response to the reviewers' comments. Your revised manuscript is also likely to be sent to reviewers for further evaluation.

When you are ready to resubmit, please upload the following:

[1] A letter containing a detailed list of your responses to the review comments and a description of the changes you have made in the manuscript. Please note while forming your response, if your article is accepted, you may have the opportunity to make the peer review history publicly available. The record will include editor decision letters (with reviews) and your responses to reviewer comments. If eligible, we will contact you to opt in or out.

[2] Two versions of the revised manuscript: one with either highlights or tracked changes denoting where the text has been changed; the other a clean version (uploaded as the manuscript file).

Important additional instructions are given below your reviewer comments.

Please prepare and submit your revised manuscript within 60 days. If you anticipate any delay, please let us know the expected resubmission date by replying to this email. Please note that revised manuscripts received after the 60-day due date may require evaluation and peer review similar to newly submitted manuscripts.

Thank you again for your submission. We hope that our editorial process has been constructive so far, and we welcome your feedback at any time. Please don't hesitate to contact us if you have any questions or comments.

Sincerely,

Marc Robinson-Rechavi
Academic Editor
PLOS Computational Biology

Ilya Ioshikhes
Section Editor
PLOS Computational Biology

****

Reviewer's Responses to Questions

Comments to the Authors:
Please note here if the review is uploaded as an attachment.

Reviewer #1: The authors investigate cell type deconvolution, where populations of cell types and/or their gene expression signatures are inferred from bulk RNA-seq samples. This investigation involves comparing multiple existing tools to the simple machine learning/statistical baselines of LASSO and Ridge regression. This topic is important and the paper is very well written. Overall, I think that the work is interesting and worth publishing but that the description could be expanded at some key points to ensure that the audience follows the precise steps taken and evaluations presented.

(1) Are the correlations for sample-level cell-type expression prediction being displayed over the same genes for each method or over different sets of genes? The authors indicate that CIBERSORTx focuses on only a subset of genes and other methods may also not make predictions for the same set of genes. It is challenging to compare methods if the performance is measured on different outputs.

Currently, the results are indeed over different sets of genes. We have now added an additional comparison restricting to the subset of genes common to all methods (S3 Fig). We find broadly the same ordering of method by performance and add the following paragraph.

> *"Because different methods imputed values for different genes, we also compared methods restricting to the common gene sets predicted by all methods and found broadly the same ordering of methods by performance (S3 Fig)."*

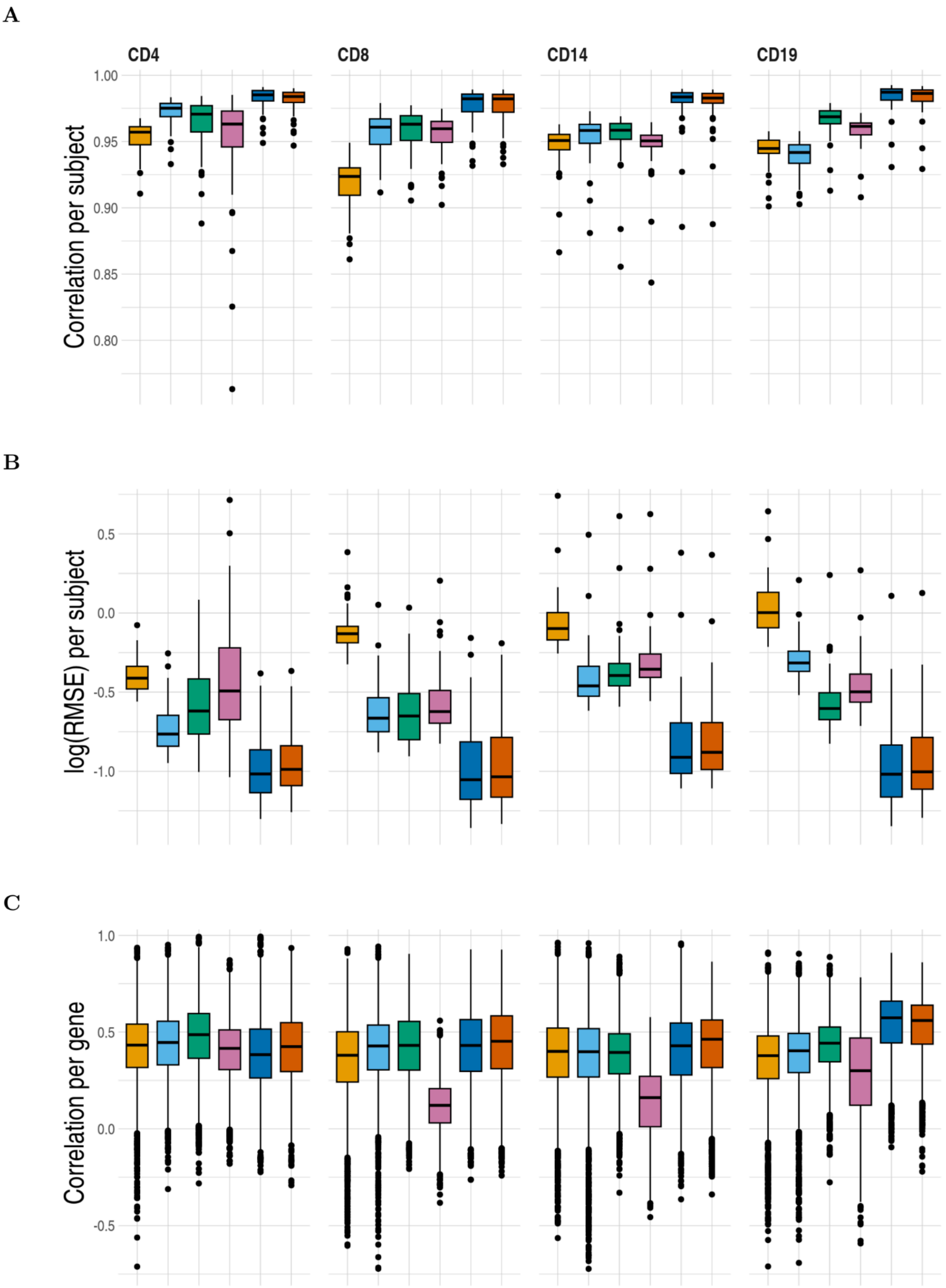
A
CD4
CD8
CD14
CD19
Correlation per subject
1.00
0.95
0.90
0.85
0.80
B
log(RMSE) per subject
0.5
0.0
-0.5
-1.0
C
Correlation per gene
1.0
0.5
0.0
-0.5

D

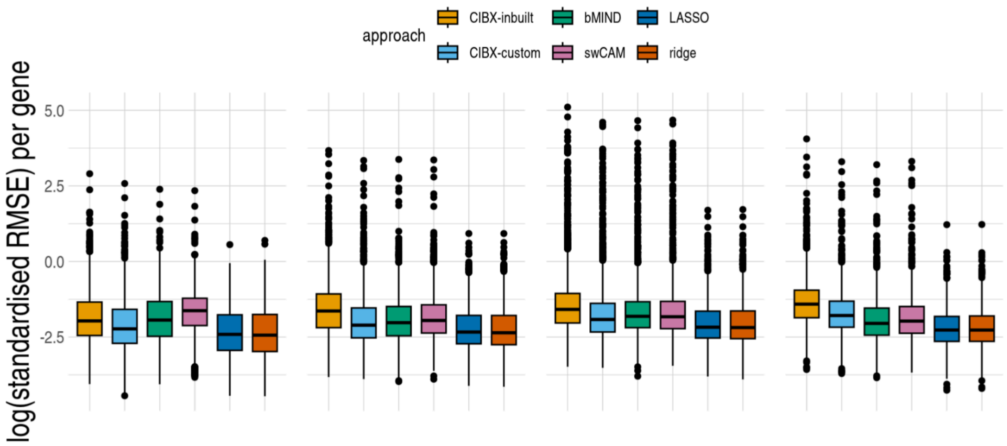

S3 Fig. Prediction accuracy of sample-level cell-type expression by approach. **For each cell type, genes common across approaches were used. No. common genes are 3837 for CD4, 6274 for CD8, 5185 for CD14, and 2689 for CD19, respectively.** (A) Pearson correlation and (B) log root mean square error (RMSE) comparing observed to predicted cell-type expression of genes from the same subjects, one estimate per subject. (C) Pearson correlation and (D) log RMSE between observed and predicted cell-type expression across testing samples for each gene, estimate per gene. RMSE was standardised by the average observed expression per gene. CIBX-inbuilt: CIBERSORTx expression deconvolution with the inbuilt signature matrix; CIBX-custom: CIBERSORTx expression deconvolution with a custom signature matrix derived from sorted cell-type expression in training samples; bMIND: bMIND expression deconvolution with flow fractions; swCAM: swCAM deconvolution with flow fractions; LASSO/ridge: expression predicted from regularised multi-response Gaussian models.

(2) It was unclear to me why the "baseline" correlation was between the *observed* expression in one individual versus the *predicted* expression in another individual. It is true that this demonstrates some level of variance across individuals but, in my mind, the baseline here is compare the average gene expression profiles in training set individuals (for each cell type) versus the gene expression profiles in test set individuals (in matched cell types). Basically, if one did not use a machine learning model, how well could one do.

We have added an additional baseline, correlations between observed expression in different subjects in matched cell types, to the original Supplementary Figure 8 (S2 Fig now). We have revised the paragraph as follows.

> *"To better interpret the correlations, we calculated "baseline" correlations. These were estimated either between observed expression in one individual and estimated expression in the same cell type from a different individual, or between the observed expression across different subjects within the matched cell types. These were also high, and only marginally different from baseline correlations, limiting the utility of this measure to discriminate among methods (S2 Fig)."*

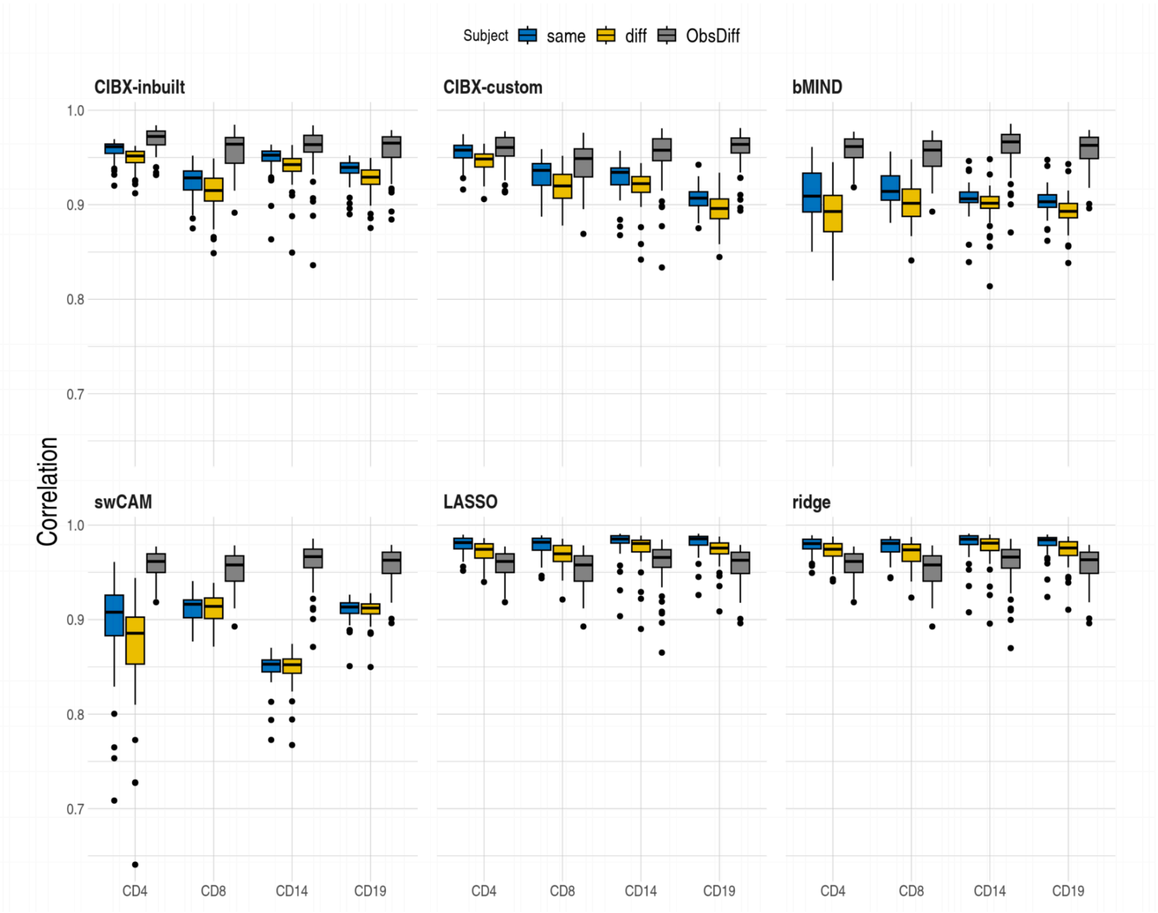

S2 Fig. Distributions of Pearson correlations (y-axis) between observed and imputed expression across genes from the same/different (diff) subjects and correlation between observed expression in different individuals in matched cell types (ObsDiff) by cell type and approach. One estimate per subject. CIBX-inbuilt: CIBERSORTx with the inbuilt signature matrix; CIBX-custom: CIBERSORTx with a custom signature matrix derived from sorted cell-type expression in training samples; bMIND: bMIND with flow fractions; swCAM: swCAM with flow fractions; LASSO/ridge: regularised multi-response Gaussian models.

(3) Are the correlations for the sample-level cell-type expression prediction calculated on the raw counts or the log2 transformed data? The sections describing the methods indicate that the models are trained to predict log2 transformed data but the performance measurement section does not specify. Given the heteroskedastic nature

of gene expression values, the more important comparison is likely on log2 transformed data.

Correlations are calculated on the log2 transformed expression. We have added "log2 transformed" to the statement below for clarity.

> *"We calculated correlations and RMSE per gene between imputed and observed log2 transformed expression to evaluate and benchmark the predicted accuracy of expression among CIBERSORTx using custom and inbuilt, bMIND using custom, swCAM with cell-type specific markers, LASSO and RIDGE"*

(4) The chunking approach for LASSO/Ridge training is a bit confusing to me and it would be helpful if the paper could expand on what precisely is being done. The sentence beginning "Specifically, we performed a hierarchical clustering.." is a bit vague. Are the cell types in the training data or are the genes in the training data? Are expression values predicted for individual genes or just for these chunks and each gene is assumed to have the same expression? The main text says "Finally, we trained regularised... models to predict cell-type expression using all genes." That statement doesn't seem correct if you are only using these chunks. It is true that you use gene expression values from all genes to derive the chunks but the models themselves only see these chunks, is that correct? Can the cross-validation of models be explained in more detail with respect to what the exact training, validation, and test sets are?

We have clarified our description of the multi-response regression models in the Methods:

> *"Standard penalised regression approaches aim to predict a single variable given observations of multiple predictors. While ordinary least squares aims to identify β that minimises $\sum_{i}^{N}(y_i - \beta_0 - x_i\beta)^2$ for predictors x_i and outcomes y_i, penalised regression approaches do this subject to constraints: $\sum_j |\beta_j| \leq \alpha$ (LASSO) or $\sum_j \beta_j^2 \leq \alpha$ (ridge). We could train a separate LASSO model using all genes in the mixed cell data to predict each gene in a sorted cell in turn. However, given the correlations between expressions of different genes, we hypothesised that multi-response models may be more accurate given they allow sharing of information between the genes to be predicted. In a multi-response model, all genes in the mixed cell data are still used but now prediction is made for a set of genes. Information is shared a coefficients for any predictor are either zero for all target genes or non-zero for all target genes. However, note that non-zero coefficients have distinct values for each target gene. Thus multi-response models are appropriate to predict subsets of correlated genes.*
>
> *We first used* cell-type specific *expression in training samples to define these subsets of correlated genes.* We clustered genes into chunks on the basis of their expression profiles (in log2 TPM), with a size of up to 500 *[… description of this unchanged …] The numbers of chunks (sizes) in the CLUSTER data were 74 (49-481) for CD4, 71 (71-493) for CD8, 76 (76-496) for CD14, and 83 (41-486) for CD19, respectively. In 24 pseudo-bulk datasets, the total numbers of gene chunks (respective size ranges) were 1358 (4-500) for CD4, 1306 (8-498) for CD8, 1533 (3-499) for CD14 and 1180 (2-497) chunks for CD19.*
>
> *For each chunk, we fitted a penalised multi-response linear regression (LASSO/ridge) performed using PBMC expression in all genes to predict cell specific expression in the chunk genes, on the log2(TPM+1) scale. [… further description unchanged …]"*

Reviewer #2: Wei-Yu Lin et al present a comparative assessment of methods to impute cell-type specific gene expression profiles from bulk RNA-seq samples, including state of the art methods and two non-domain specific regression models (LASSO and RIDGE). They provide a summary of the state of the art methods and different metrics to evaluate the performance.

I believe that the comparative assessment is relevant to the field, and the community

would benefit from an unbiased analysis of existing methods. However, in order to be considered for publication I believe the manuscript needs to be improved under different points of view, that I highlighted in this review.

Abstract and author summary:
I think that the author summary is much more clear than the abstract. I would personally suggest to rewrite the abstract with the same information flow followed in the author summary.
In fact, starting the first sentence in the abstract mentioning DGE might be misleading, since it suggests that the paper addresses the problem of inferring DGE.
Instead, in the author summary the addressed problem is explained and motivated, and the impact on DGE inference is subsequently addressed, and this makes it easier for the reader to grasp the concepts presented in the article.

We have restructured the abstract so that DGE inference only appears in the final paragraph. We agree this is a clearer summary of the work.

> *"Gene expression studies often use bulk RNA sequencing of mixed cell populations because single cell or sorted cell sequencing may be prohibitively expensive. However, mixed cell studies may miss expression patterns that are restricted to specific cell populations. Computational deconvolution can be used to estimate cell fractions from bulk expression data and infer average cell-type expression in a set of samples (eg cases or controls), but imputing sample-level cell-type expression is required for more detailed analyses, such as relating expression to quantitative traits, and is less commonly addressed.*
>
> *Here, we assessed the accuracy of imputing sample-level cell-type expression using a real dataset where mixed peripheral blood mononuclear cells (PBMC) and sorted (CD4, CD8, CD14, CD19) RNA sequencing data were generated from the same subjects (N=158), and pseudobulk datasets synthesised from eQTLgen single cell RNA-seq data. We compared three domain-specific methods, CIBERSORTx, bMIND and debCAM/swCAM, and two cross-domain machine learning methods, multiple response LASSO and ridge, that had not been used for this task before.*
>
> *We also assessed the methods according to their ability to recover differential gene expression (DGE) results. LASSO/ridge showed higher sensitivity but lower specificity for recovering DGE signals seen in observed data compared to deconvolution methods, although LASSO/ridge had higher area under curves than deconvolution methods. Machine learning methods have the potential to outperform domain-specific methods when suitable training data are available."*

Introduction:
The introduction needs to be re-written with a more clear and schematic flow.
- Authors make a distinction between methods that work with f and m vectors and those that infer M and F as matrices for multiple samples. If it is necessary for understanding the addressed task, authors should start by describing the two settings more clearly, and they should clarify which tools work with multi-sample matrices and which ones infer f and m vectors for one sample.

We begin by introducing the deconvolution equation that is central to many methods as m = H x f using vector notation (one individual), as that is simplest to understand. But writing it in matrix notation doesn't distinguish between methods, it is just convenience when dealing with a set of samples instead of a single individual.

- Authors should also clarify which methods keep f or H fixed, and which ones infer both of them simultaneously.

We have tried to clarify various parts of the introduction, also taking into account your later comments on mathematical notation and the distinction between H and H1. Our revised introduction distinguishes between H, H1 and G (a new introduction):

> *"Note that while our goal here is to impute cell type specific expression profiles for each individual, H is often treated as constant across a population, or some subgroup of the population (written H1). The sample-level cell-type expression profiles for a cohort must be indexed by individual, and may be written as G (n × c × k) where k is the number of samples."*

We hope that this distinction clears up much of the confusion below. We have introduced G for individual-level cell specific expression profiles, because this makes it a lot easier later to distinguish between, for example, CIBERSORTx which imputes expression across all cells and individuals for each gene in parallel

> *"CIBERSORTx iteratively applies bootstrapped NNLS to estimate and refine cell-type expression coefficients for imputing $G_{i,\bullet,\bullet}$ based on solving the equation $G_{i,\bullet,\bullet} \times F = M_{i,\bullet}$ where F is first estimated as above."*

and the penalised regression methods which impute expression for all genes and individuals for each cell type in parallel

> *"they can be used to estimate $G_{\bullet,j,\bullet}$ for each cell type j=1,...,c independently given training data - samples in which both cell type specific and mixed cell transcriptomes have been measured. "*

- In my opinion the description of methods such as Rodeo and csSAM, which are not involved in the comparative assessment, should be removed.

Rather than remove, we have instead listed them without details on their methods, to indicate that CIBERSORTx is among a set of methods that are available to estimate H1.

- Finally I would make a figure with a summary of the methods, indicating what values are inferred and what approach is employed by each of the presented tools.

We have summarised the matrices estimated and the approaches utilised by each method in new S1 Table and referred to it in the the paragraph below

> *"We identified five out of ten existing methods (S1 Table) that have developed strategies to impute sample-level cell-type gene expression:"*

S1 Table. Summary of existing deconvolution approaches

Approach/matrices	signature genes H	cell fractions F	average cell-type expression $H1$	sample-level cell-type expression G	evaluation in this study
CIBERSORTx	DGE[a]	ν vector regression	NNLS	NNLS	Yes
bMIND	x	NNLS	BLMM	BLMM	Yes
debCAM/swCAM	OVE.FC[b]	pcaPP::l1median[b]	NNLS	LMM	Yes
MIND	x	NNLS	EB-GLM[c]	EB-GLM[c]	No
CellR	x	LP[d]	x	SA[d]	No
Rodeo	x	x	NNLS	x	No
csSAM	x	x	NNLS	x	No
EPIC	x	NNLS	x	x	No
quanTIseq	x	NNLS	x	x	No
FARDEEP	x	NNLS	x	x	No

NNLS: non-negative least squares, noted that NNLS implementation differ by approach; BLMM: Bayesian mixed-effects model

[a] differential gene expression (DGE) analysis of purified cell populations

[b] Genes with fold changes (FC) over the predefined threshold in the comparisons of one verse everyone (OVE) cell populations; pcaPP::l1median: spatial median projection; LMM: linear mixed model

[c] EB-GLM: empirical Bayes generalised linear model and it requires multiple RNA-seq expression measurements per subjects

[d] LP: linear programming; simulated annealing (SA) algorithm and it requires predefined cell-type clusters in scRNA-seq data

Mathematical notation: I think that the mathematical notation needs to be more thorough.
- Why on page 5 H became referred to as H1?

See above

- I may have not understood some key element in the paper, but I am finding it difficult to understand from the manuscript what matrix is inferred by CIBERSORTx: if the signature matrix H is provided as input, what are the expression values inferred by the tools? I think that this could be clarified with a more thorough mathematical notation to describe each task performed by the methods in the comparison.
For example, authors could employ a similar description as that presented in CIBERSORTx methods, where it is explicitly reported that the imputed gene expression matrix is derived by first inferring the abundance vector F using a signature matrix B containing a subset of markers, and then solving the system of linear equations M = H x F.

See above

- Overall, I think it is necessary to use a specific mathematical notation for each concept discussed in the manuscript: if H is the imputed gene expression matrix, which is

different from the signature matrix (denoted with B in CIBERSORTx manuscript, methods, page 11 of the PDF), this signature matrix needs to be explicitly described.

See above

- The two regression methods (LASSO and RIDGE) analysed need to be more systematically described. Given that they have not been presented in the context of gene expression imputation before, authors need to provide a solid mathematical representation of the regression models.

We are not convinced that systematic description of LASSO or ridge would be useful, as they are now quite standard techniques. Instead, we added a single sentence that summarises the distinction from OLS.

> *"While ordinary least squares aims to estimate* β *that minimises* $\sum_{i}^{N}(y_i - \beta_0 - x_i\beta)^2$ *for predictors* x_i *and outcomes* y_i*, penalised regression approaches do this subject to constraints:* $\sum_j |\beta_j| \leq \alpha$ *(LASSO) or* $\sum_j \beta_j^2 \leq \alpha$ *(ridge)."*

- A graphical notation about the matrices could be added in the same figure summarising the compared methods.

See above (new S1 Table)

- Authors should write explicitly in the methods the vectors and formulas used to calculate the performances, keeping the notation consistent with that adopted in the description (M = H x F)

See above

- The simulations of phenotypes for the DGE analysis needs to be explained more clearly.

We have added sentences on how simulated phenotypes were generated and revised the following paragraph.

> *"We simulated ten phenotypes that correlated with gene expression in the observed cell type data, including testing samples, using principal component analysis (PCA). First we conducted PCA of the fully observed gene expression data in the target cell type. From each of these we derived a continuous and a binary phenotype. The continuous phenotypes were defined as the PC value plus a standard normal error. The binary phenotypes were by dichotomising each PC with values >0 designated as 1 for pseudo-cases; otherwise, 0 for pseudo-controls. For each simulated phenotype, DGE analysis…"*

Comparative assessment results:
- Regarding the LASSO and RIDGE regression, in order to state that they provide better estimates of imputed gene expression values I think it is fundamental to test them on external datasets that are not related to those employed in the CLUSTER project.
- For a more extensive comparison of the tools, authors should include tests on synthetic data. One possibility is to use a single-cell RNA sequencing simulation tool such as Splatter, which can estimate simulations parameters from real data and can thus provide simulated cells consistent with real datasets.

Single cell counts can be aggregated to produce a pseudo-bulk expression vector per sample, which can be used to test the different methods discussed in the manuscript.

We have added an additional dataset, using aggregated single cell counts to produce pseudo-bulk as you suggest. We have evaluated the performance using these pesudobulk datasets and found better DGE recovery of LASSO/ridge than other deconvolution-based methods. We have incorporated this finding to the result section in the following paragraph

> *"To provide a broader range of scenarios to compare these methods, we constructed cell-specific bulk RNA-seq from single cell data from eQTLgen [27] and performed a similar analysis. We compared expression between cells stimulated with C. albincas for 3 hours to untreated cells, in three settings: either unadjusted for batch, adjusted using batch as a covariate, or adjusted using Combat-seq This allowed us to consider different models including covariates or different ways to account for batch effects. We varied training sample size from 20 (25%) to 80 (100%) samples. As the training sample size increased, AUCs generally increased for all approaches. LASSO and ridge regression consistently outperformed other methods across all cell types, regardless of whether raw or batch-corrected DGE results, in which batch effects were accounted for as a covariate or batch-corrected read counts by Combat-seq were used (Fig 5). This dominance persisted in further analysis of common gene sets (S8 Fig). All our results, taken together, suggest that regularised multivariate models performed better than the other three deconvolution-based methods."*

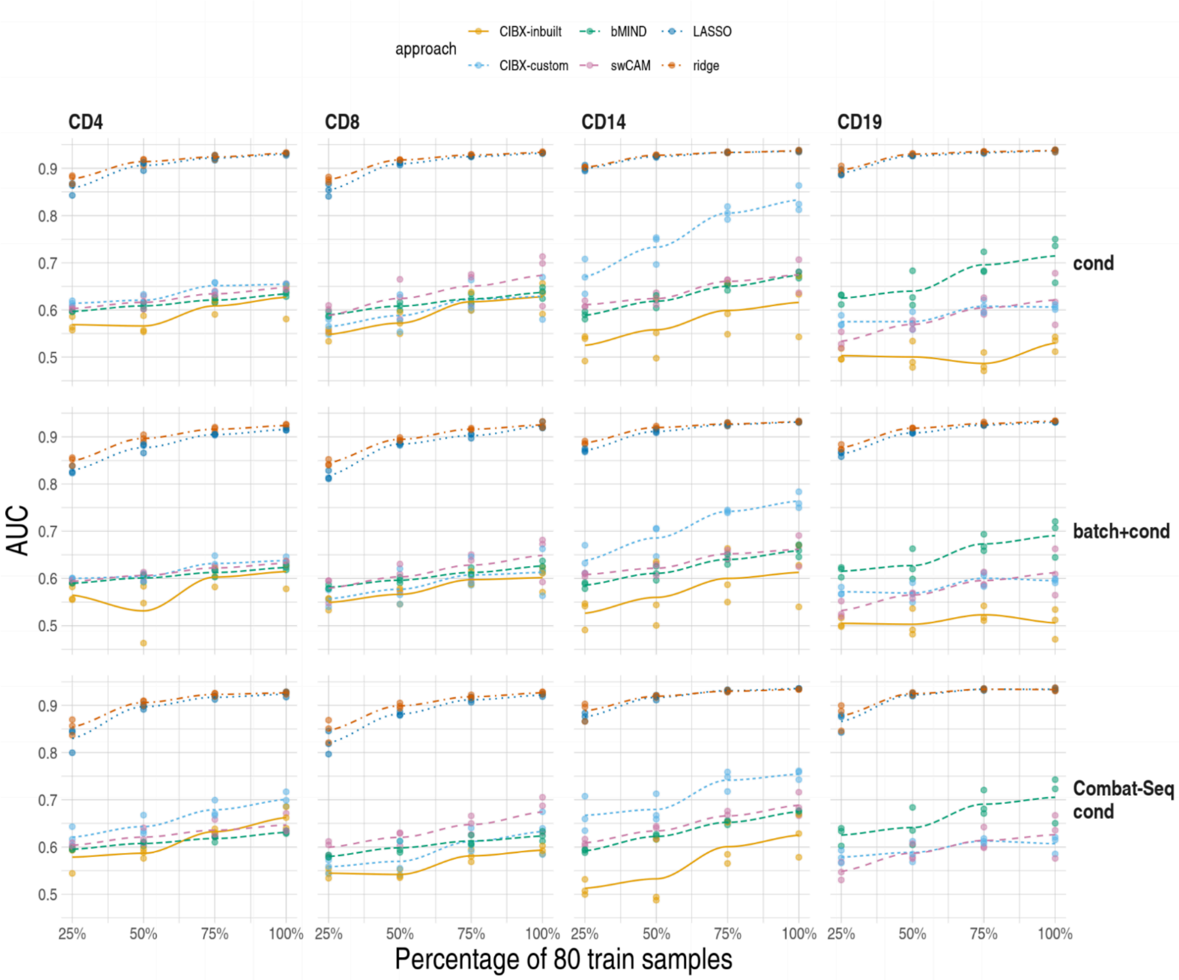

Fig 5. Differential gene expression (DGE) recovery based on pseudobulk data. We varied training sample size from 20 (25%) to 80 (100%) (x-axis) and quantified area under curve (AUC) for DGE recovery (y-axis) by cell type (columns) for each scenario (rows). cond: raw aggregated read counts were used for DGE analysis of in vitro stimulation with C. albicans after 3 hours (3hCA) vs untreated (UT). batch+cond: same as cond, and with batch (V2 & V3 chemistry) as a covariate in the 3hCA vs UT DGE model. Combat-seq cond: Combat-seq batch-corrected read counts were used in DGE analysis of 3hCA vs UT. Each point is the result of one analysis, and three replicates were conducted for each training sample size. For each pseudobulk data, the receiver operating characteristic curve and AUC were estimated by FDR fixed at 0.05 in the observed DGE results and varied FDRs from 0 to 1 by 0.05 in the DGE results using imputed expression. Local polynomial regression fitting (loess) were plotted for each approach. Note that CIBX-inbuilt is not shown for CD19/Combat-Seq cond. This is because it was able to impute < 60 genes, compared to $\sim$ 1,000 for CIBX-custom and $>$ 11,000 for other methods, so estimates of AUC are very noisy. CIBX-inbuilt: CIBERSORTx with the inbuilt signature matrix; CIBX-custom: CIBERSORTx with a custom signature matrix based on pure cell expression in the training samples; bMIND: bMIND with true fractions; swCAM: swCAM with true fractions; LASSO/ridge: regularised multi-response Gaussian models.

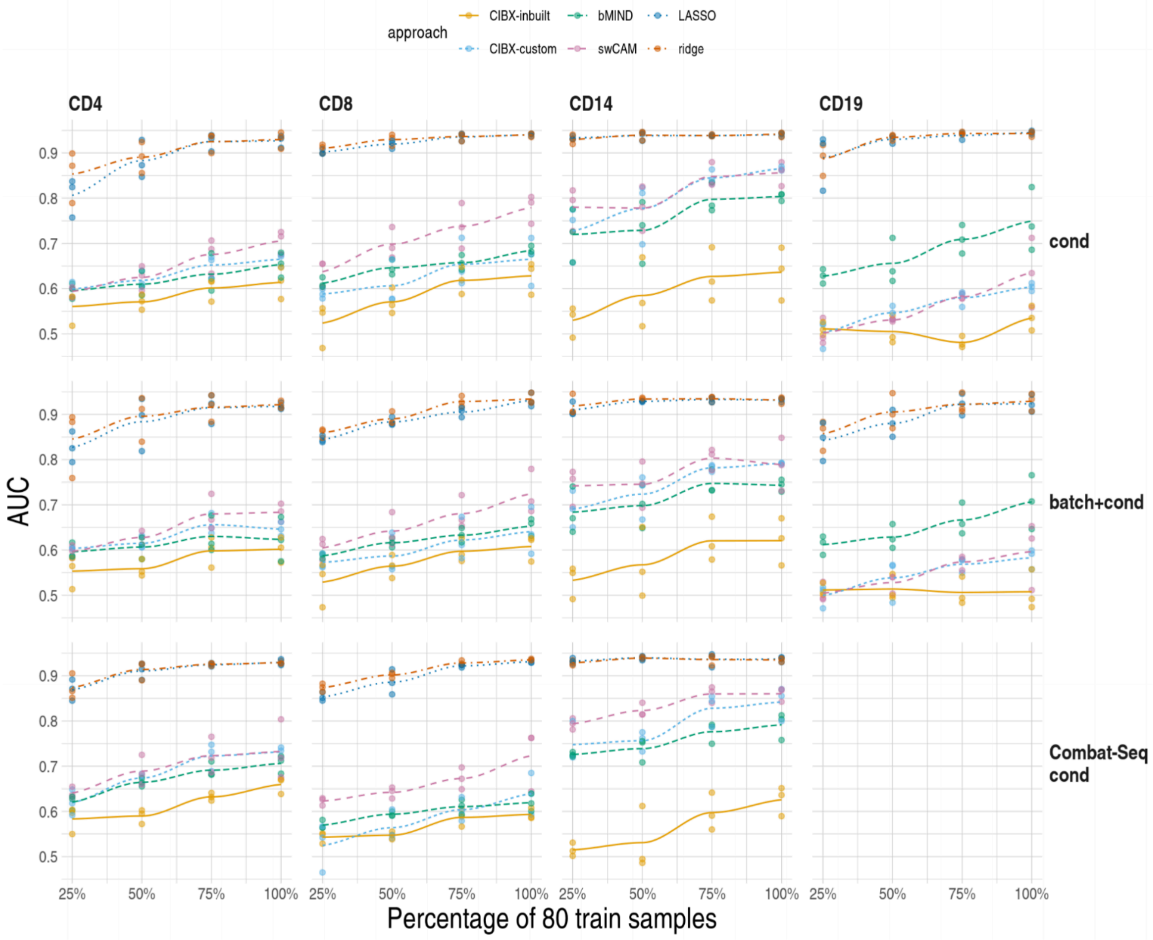

S8 Fig. Differential gene expression (DGE) recovery based on pseudobulk data. We varied training sample size from 20 (25%) to 80 (100%) (x-axis) and quantified area under curve (AUC) for DGE recovery (y-axis) by cell type (columns) for each scenario (rows). cond: raw aggregated read counts were used for DGE analysis of in vitro stimulation with C. albicans after 3 hours (3hCA) vs untreated (UT). batch+cond: same as cond, and with batch (V2 & V3 chemistry) as a covariate in the 3hCA vs UT DGE model. Combat-seq cond: Combat-seq batch-corrected read counts were used in DGE analysis of 3hCA vs UT. DGE analysis was performed in **the training and testing samples** and restricted to **common gene sets predicted by all methods**. Each point is the result of one analysis, and three replicates were conducted for each training sample size. For each pseudobulk data, the receiver operating characteristic curve and AUC were estimated by FDR fixed at 0.05 in the observed DGE results and varied FDRs from 0 to 1 by 0.05 in the DGE results using imputed expression. Local polynomial regression fitting (loess) were plotted for each approach. **Noted that no data is shown for CD19/Combat-Seq cond because the common gene set predicted by all methods equals zero.** CIBX-inbuilt: CIBERSORTx with the inbuilt signature matrix; CIBX-custom: CIBERSORTx with a custom signature matrix based on pure cell expression in the training samples; bMIND: bMIND with true fractions; swCAM: swCAM with true fractions; LASSO/ridge: regularised multi-response Gaussian models.

- Authors should also provide a better analysis of the computational time and memory usage of the methods, varying the number of subpopulations and the number of samples.

We have added an additional S9 Fig, which uses analysis of the pseudo bulk data above to show that CPU time scales linearly with sample size and memory usage stays fairly constant across sample sizes.

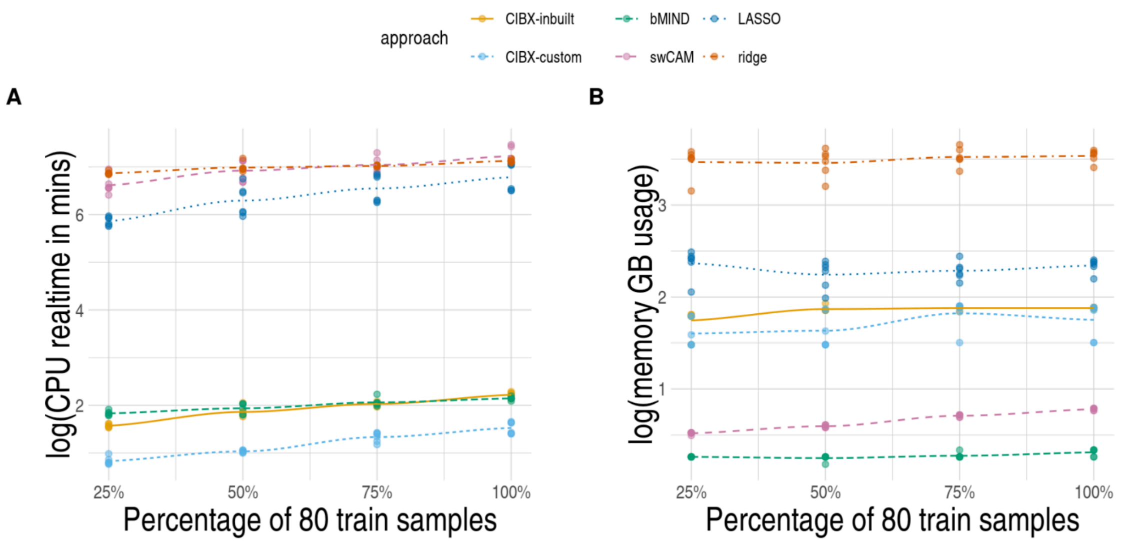

S9 Fig. Resource metrics based on pseudobulk data (A) CPU time in minutes (B) memory GB usage in the nature logarithm scale (y-axis) across 25%, 50%, 75% and 100% percentage of 80 train samples (x-axis). For serial jobs, swCAM, LASSO and ridge, CPU time was summed together for the same pseudobulk data, and median of memory usage was taken per pseudobulk data. Each point is a pseudobulk data. Local polynomial regression fitting (loess) were plotted for each approach. CIBX-inbuilt: CIBERSORTx with the inbuilt signature matrix; CIBX-custom: CIBERSORTx with a custom signature matrix generated using pure cell expression of the training samples in the pseudobulk data; bMIND: bMIND with true fractions; swCAM: swCAM with true fractions; LASSO/ridge: regularised multi-response Gaussian models.

Reviewer #3: Lin et al. present an interesting manuscript focused on cell-type specific expression imputation and show that one-step ML models perform better for this task than the otherwise typically applied deconvolution-based approaches on their dataset. I think this is a relevant paper and fits the scope of PLOS Comp. Bio in general. I have some concerns about reproducibility and computational things, which should be easily remediable.

Otherwise, the dataset the authors use is extremely interesting and important to the research community, but I am not including it in my review here, as it is, from what I understand, not claimed as a contribution of this particular paper.

My other major concern(s) center around downstream tasks of cell-type specific expression imputation. First, I think the authors should either present some downstream results of their proposed methods (i.e., Ridge/Lasso for cell-type specific expression imputation) on non-simulated data to highlight the biological relevance of their contribution or lean more heavily into the methodological side, which for me would mean additional datasets/simulations as well as baselines. In either case, I would appreciate further comments on relevant downstream tasks of cell-type specific expression

imputation - while of course, the raw imputation is already very interesting methodologically, the authors focus on DGE as a downstream task, in which case I do not understand off the bat why their methods cannot also be compared to cell-type specific DGE methods and benchmarks [1, 2, 3]. There is mention of quantitative traits and/or covariates, hinting, I think, that some of the methods (such as [2] or csSAM) are restricted to a two-group comparison setting. I don't see how the DGE recovery downstream task actually encapsulates this, however? If it accounts for covariates, it would be useful to emphasize this and perhaps still make a limited comparison, either by restricting all methods to the two-group setting (since I understand all simulated phenotypes are binary) and/or ignoring other covariates.

Otherwise I found the paper rigorous in that no overly grandiose claims were made, and weaknesses of the proposed methods (in particular strong reliance of the ML methods on [large-ish] training datasets which may not always be available) were acknowledged.

I summarize my comments overall below as follows:
Major:
- I like that the authors have paid attention to computational considerations, but have some concerns regarding these. (i) To what extent were different methods parallelized? I see that e.g., for glmnet, a parallel backend was registered, whereas e.g., for bMIND I see no mention of parallelization. Please clarify this - IMO, if some of the methods cannot be parallelized (adequately), all methods should be compared unparallelized for fairness.

We take a different view. If a method which has been carefully parallelised is compared to one which has not, we do not think it is really fair to penalise the parallelised method. On the other hand, we agree it is very important to be explicit about the number of cores made available for each method, and we have now done that in S2 Table.

S2 Table. Computational time and memory usage by approach based on the CLUSTER data

approach	No. CPUs	CPU time (minutes)	memory usage (Gb)	No. chunks[b]	CPU time chunks[c] Q50 (Q25, Q75)
CIBX-inbuilt	8	8.80	11.0	-	-
CIBX-custom	8	4.45	5.5	-	-
bMIND	4	12.00	1.8	-	-
swCAM	10/1[a]	1713.13[b]	2.95[b]	-	-
LASSO	1	624.22[b]	16.7[b]	304	1.84 (1.23,2.73)
ridge	1	2928.45[b]	58.5[b]	304	8.85 (6.09,12.78)

No.CPUs: CPUs assigned; memory usage: physical RAM memory and swap disk space. Q50 (Q25, Q75): median (25%,75% quantiles); - : not applicable

[a] 10 CPUs for cross-validation and 1 CPU for predicting sample-level cell type expression

[b] Serial jobs: CPU time was summed together, and the median memory usage between jobs was displayed

[c] LASSO and ridge only

(ii) Why are all numbers integers in Supplementary Table? E.g., the RAM for Ridge and Lasso looks unbelievably high if it's really in GB.

We rounded the numbers to integers, and in the original Supplementary Table 1 (now S2 Table), we have rounded them to one or two decimal places. Yes, the values are GB, but we had erroneously added memory usage over the multiple chunks used (each set of correlated genes predicted is run as a separate chunk). As we already added CPU time over the chunks, the memory usage should be reported per CPU, which is what we do in the revised table. We have added these details in the table's footnote for clarity.

(iii) Ideally, the authors should rerun the methods multiple times to evaluate their computational features and indicate mean +- standard deviation or similar.

We have rerun the methods on multiple datasets synthesised from single cell data (N=24) and summarised resource metrics, with median and 25% and 75% quantiles in S3 Table.

S3 Table. Computational time and memory usage by approach based on the eQTLgen pseudobulk data

approach	No. datasets	No. CPUs	CPU time (minutes)	memory usage (Gb)	No. chunks[c]	CPU time chunks[c] Q50 (Q25, Q75)
CIBX-inbuilt	24	4	7.22 (5.64,8.12)	6.45 (6.25,6.6)	-	-
CIBX-custom	24	4	3.08 (2.71,4.08)	6 (4.47,6.53)	-	-
bMIND	24	4	7.61 (6.33,8.18)	1.3 (1.3,1.3)	-	-
swCAM	24	10/1[a]	1103.68 (799.36,1256.04)[b]	1.92 (1.77,2.07)[b]	-	-
LASSO	24	1	592.13 (391.41,862.2)[b]	10.4 (9.46,10.94)[b]	5377	1.88 (0.54,3.85)
ridge	24	1	1103.6 (1017.01,1199.88)[b]	33.6 (32.83,35.35)[b]	5377	4.62 (2.1,7.25)

No.CPUs: CPUs assigned; memory usage: physical RAM memory and swap disk space; Numbers in CPU time and memory usage indicate median (25%, 75% quantiles) across 24 pseudobulk data

[a] 10 CPUs for cross-validation and 1 CPU for predicting sample-level cell type expression

[b] Serial jobs: CPU time was summed together, and the median memory usage between jobs was taken on a pseudobulk data basis.

[c] LASSO and ridge only; No.chunks: total chunks across 24 pseudobulk data. CPU time was summarised in a chunk-level

(iv) Why is CiberSortX inbuilt missing from Supplementary Table 1?

CIBX-inbuilt predicted 11 cell-type specific expression on the sample level, so we felt comparing its resource usage to those from the methods predicting expression for 4 cell types is not really fair. For completeness, we have added CIBX-inbuilt (CiberSortX inbuilt) in the Supplementary Table (now S2 Table).

S2 Table. Computational time and memory usage by approach based on the CLUSTER data

approach	No. CPUs	CPU time (minutes)	memory usage (Gb)	No. chunks[b]	CPU time chunks[c] Q50 (Q25, Q75)
CIBX-inbuilt	8	8.80	11.0	-	-
CIBX-custom	8	4.45	5.5	-	-
bMIND	4	12.00	1.8	-	-
swCAM	10/1[a]	1713.13[b]	2.95[b]	-	-
LASSO	1	624.22[b]	16.7[b]	304	1.84 (1.23,2.73)
ridge	1	2928.45[b]	58.5[b]	304	8.85 (6.09,12.78)

No.CPUs: CPUs assigned; memory usage: physical RAM memory and swap disk space. Q50 (Q25, Q75): median (25%,75% quantiles); - : not applicable

[a] 10 CPUs for cross-validation and 1 CPU for predicting sample-level cell type expression

[b] Serial jobs: CPU time was summed together, and the median memory usage between jobs was displayed

[c] LASSO and ridge only

- I like the premise of this study but feel that to round it out, it would ideally need (i) further biological downstream analysis, using the proposed methods and/or (ii) more detailed experiments, turning it into an almost quasi-benchmark. Following on (i), the paper mentions "The CLUSTER Consortium aims to use immune cell RNA-seq data to find transcriptional signatures which predict treatment response in childhood arthritis.". I think the paper could be much stronger if the authors could give e.g., some examples they can discover downstream in their data by using their proposed approach with Ridge or Lasso that would not have been discoverable with the other compared to deconvolution approaches.

We cannot do this, as the main analysis of these data will be submitted to a clinical journal appropriate to the target audience, and that journal would not accept results already published elsewhere as novel. At the same time, a clinical journal would not be the right place to describe this comparison of methods, since it would not reach the target audience. Instead, we have focused on your point (ii). As described above, we analysed new pseudobulk data generated by aggregating counts from single cell data.

> *"To provide a broader range of scenarios to compare these methods, we constructed cell-specific bulk RNA-seq from single cell data from eQTLgen [27] and performed a similar analysis. We compared expression between cells stimulated with C. albincas for 3 hours to untreated cells, in three settings: either unadjusted for batch, adjusted using batch as a covariate, or adjusted using Combat-seq This allowed us to consider different models including covariates or different ways to account for batch effects. We varied training sample size from 20 (25%) to 80 (100%) samples. As the training sample size increased, AUCs generally increased for all approaches. LASSO and ridge regression consistently outperformed other methods across all cell types, regardless of whether raw or batch-corrected DGE results, in which batch effects were accounted for as a covariate or batch-corrected read counts by Combat-seq were used (Fig 5). This dominance persisted in further analysis of common gene sets (S8 Fig). All our results, taken together, suggest that regularised multivariate models performed better than the other three deconvolution-based methods."*

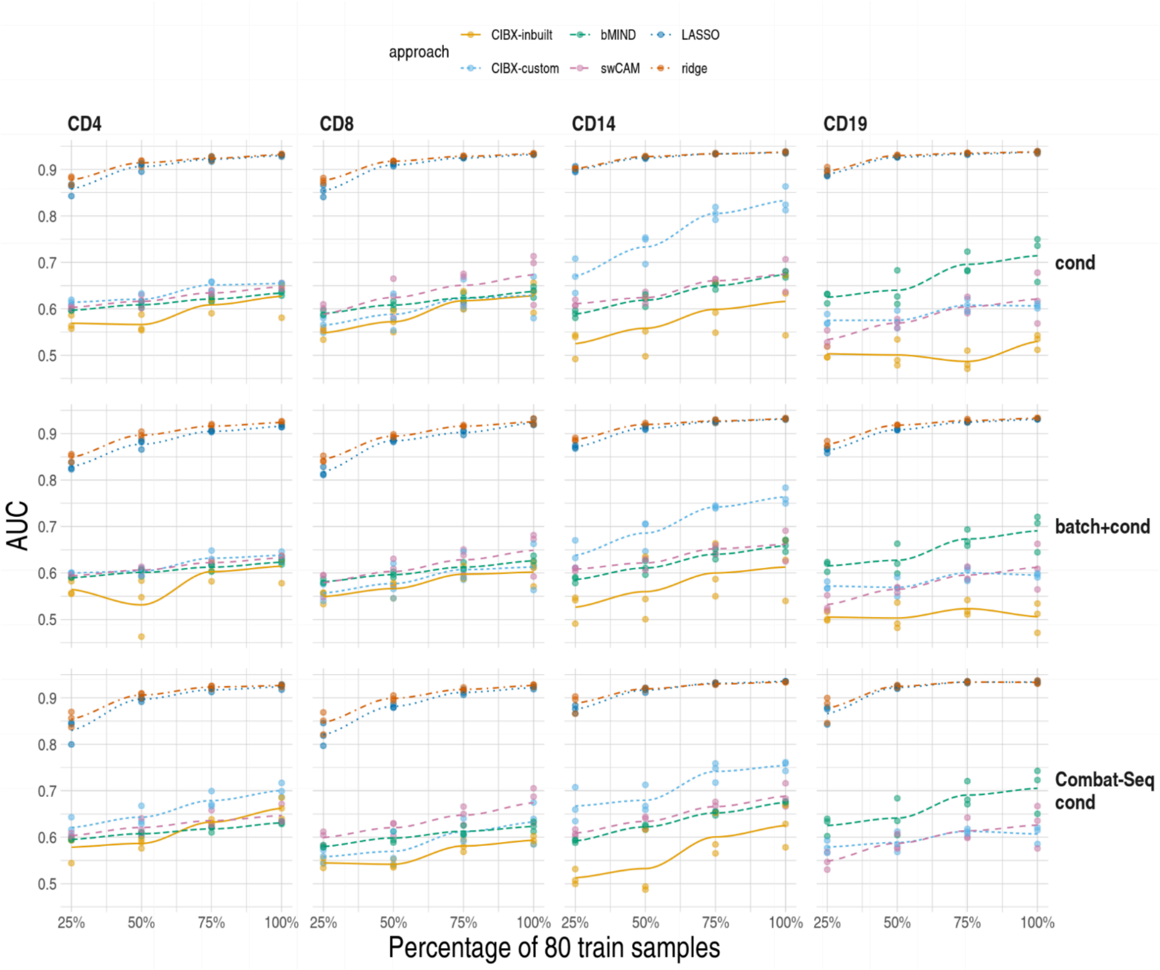

Fig 5. Differential gene expression (DGE) recovery based on pseudobulk data. We varied training sample size from 20 (25%) to 80 (100%) (x-axis) and quantified area under curve (AUC) for DGE recovery (y-axis) by cell type (columns) for each scenario (rows). cond: raw aggregated read counts were used for DGE analysis of in vitro stimulation with C. albicans after 3 hours (3hCA) vs untreated (UT). batch+cond: same as cond, and with batch (V2 & V3 chemistry) as a covariate in the 3hCA vs UT DGE model. Combat-seq cond: Combat-seq batch-corrected read counts were used in DGE analysis of 3hCA vs UT. Each point is the result of one analysis, and three replicates were conducted for each training sample size. For each pseudobulk data, the receiver operating characteristic curve and AUC were estimated by FDR fixed at 0.05 in the observed DGE results and varied FDRs from 0 to 1 by 0.05 in the DGE results using imputed expression. Local polynomial regression fitting (loess) were plotted for each approach. Note that CIBX-inbuilt is not shown for CD19/Combat-Seq cond. This is because it was able to impute $<$ 60 genes, compared to $\sim$ 1,000 for CIBX-custom and $>$ 11,000 for other methods, so estimates of AUC are very noisy. CIBX-inbuilt: CIBERSORTx with the inbuilt signature matrix; CIBX-custom: CIBERSORTx with a custom signature matrix based on pure cell expression in the training samples; bMIND: bMIND with true fractions; swCAM: swCAM with true fractions; LASSO/ridge: regularised multi-response Gaussian models.

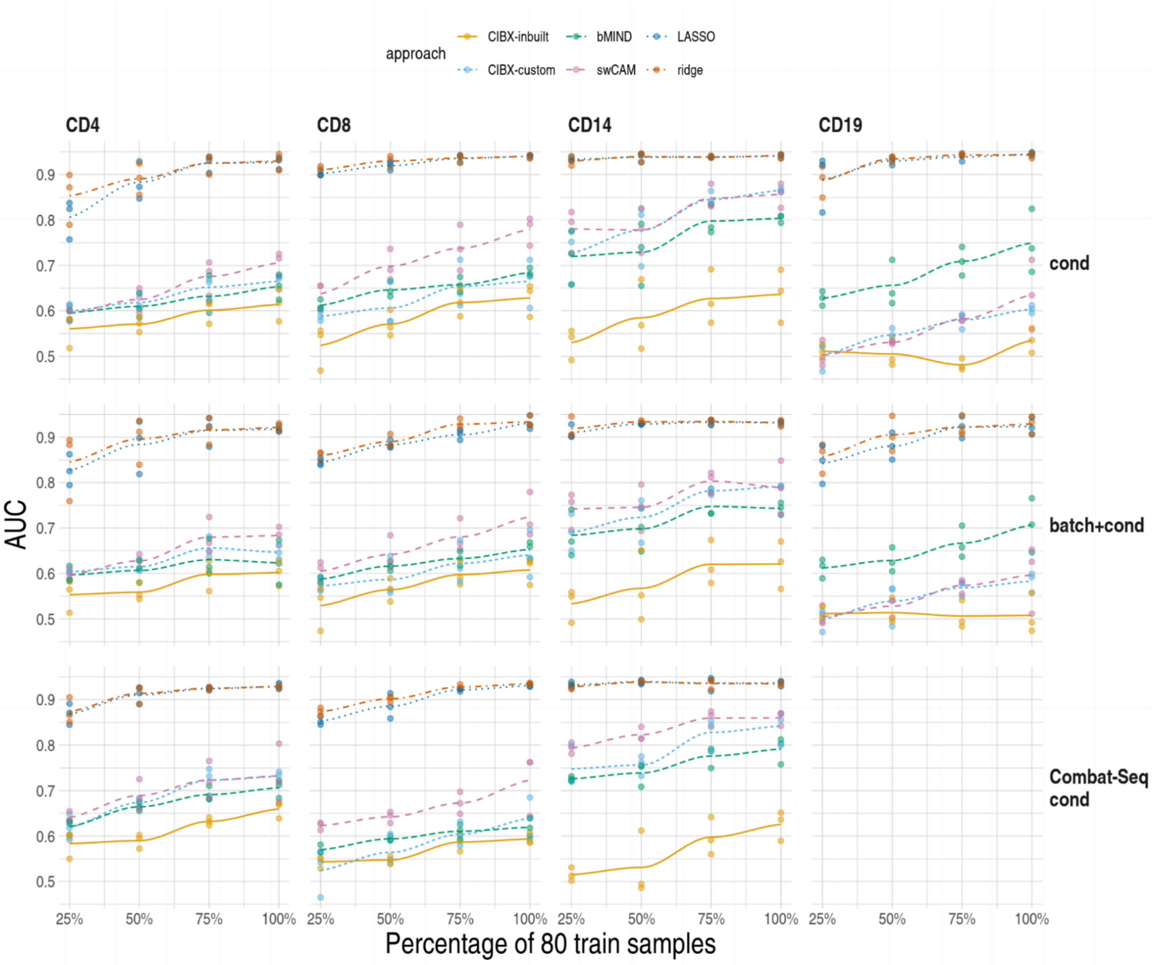

S8 Fig. Differential gene expression (DGE) recovery based on pseudobulk data. We varied training sample size from 20 (25%) to 80 (100%) (x-axis) and quantified area under curve (AUC) for DGE recovery (y-axis) by cell type (columns) for each scenario (rows). cond: raw aggregated read counts were used for DGE analysis of in vitro stimulation with C. albicans after 3 hours (3hCA) vs untreated (UT). batch+cond: same as cond, and with batch (V2 & V3 chemistry) as a covariate in the 3hCA vs UT DGE model. Combat-seq cond: Combat-seq batch-corrected read counts were used in DGE analysis of 3hCA vs UT. DGE analysis was performed in **the training and testing samples** and restricted to **common gene sets predicted by all methods**. Each point is the result of one analysis, and three replicates were conducted for each training sample size. For each pseudobulk data, the receiver operating characteristic curve and AUC were estimated by FDR fixed at 0.05 in the observed DGE results and varied FDRs from 0 to 1 by 0.05 in the DGE results using imputed expression. Local polynomial regression fitting (loess) were plotted for each approach. **Noted that no data is shown for CD19/Combat-Seq cond because the common gene set predicted by all methods equals zero.** CIBX-inbuilt: CIBERSORTx with the inbuilt signature matrix; CIBX-custom: CIBERSORTx with a custom signature matrix based on pure cell expression in the training samples; bMIND: bMIND with true fractions; swCAM: swCAM with true fractions; LASSO/ridge: regularised multi-response Gaussian models.

- Also, following up on (ii) of my previous point, it would (alternatively or in addition to the downstream biological investigation of the dataset via the newly proposed methods) be interesting to evaluate alternative baselines and/or datasets. Concretely, I wonder a bit about the main downstream tasks of imputation of cell-type specific expression. As I see it, the main downstream task (also as presented in the paper) is differential expression. In that case, however, the authors should also compare to dedicated

methods for cell-type specific differential expression. I am not an expert on this, but there seem to exist several benchmarks [1, 2] and methods [3] (see my comment above re limitations to the two-group comparison setting). I suggest the authors either clarify/expand their choice of downstream tasks and/or add methods for cell-type specific differential expression to the analysis, ideally also in other datasets (e.g., [1, 2] both have simulated datasets that could be used).

1] Jaakkola, Maria K., and Laura L. Elo. "Estimating cell type-specific differential expression using deconvolution." Briefings in Bioinformatics 23.1 (2022): bbab433.

[2] Meng, Guanqun, et al. "A comprehensive assessment of cell type-specific differential expression methods in bulk data." Briefings in Bioinformatics 24.1 (2023): bbac516.

[3] Tang, Daiwei, Seyoung Park, and Hongyu Zhao. "SCADIE: simultaneous estimation of cell type proportions and cell type-specific gene expressions using SCAD-based iterative estimating procedure." Genome Biology 23.1 (2022): 129.

*Given our motivating example is to run gene expression analysis with quantitative phenotypes, we have decided not to employ the methods 1-3, as they estimate **average** cell type specific expression in two groups and compare the estimates. Instead, we have addressed your request to add additional comparisons of the existing methods based on simulated datasets to enable a more comprehensive comparison (see above).*

Minor:
- I appreciate that the authors have made code (and processed data) available. That said, I think some things are missing from the code repo to ensure optimal reproducibility. First, it would be important to note the versions of all R packages (as well as R [I realize this is mentioned in the paper, but IMO the repo should be self-contained concerning reproduction]). If this is integrated into Nextflow (e.g., via conda/renv) even better, but this is not necessary. However, at a minimum, there should be a text file listing all package versions (effectively just having the sessionInfo separately as opposed to in the HTML where it may not be immediately visible, I mean), ideally even a renv.lock file or comparable, which makes it feasible for interested researchers to reproduce the author's results in the same environment. Second, the repo needs additional documentation as to how the results can be reproduced. In particular, it should cover these three questions: (i) Which commands should be executed to set up everything (e.g., download data, install requirements if any, ...) (ii) Which commands should be executed to rerun the experiments (iii) Which commands should be executed to reproduce the figures (from what I can tell this is already in the RMDs, but would be still worth pointing out explicitly since I didn't see this or the session info until I looked at the HTML). [In case I missed any of these, perhaps just highlight them more clearly in the repro as I guess I am somewhat close to your "average target audience" wrt reproduction]

We have provided the details on (i) how to set up everything , (ii) how to rerun the experiments, and (iii) how to generate the results in the README of the new repository (https://gitlab.com/b8307038/sceqtlgen).

- I am quite unclear about how the "simulation" for DGE recovery was performed, I suggest the authors re-check this section to make it more clear.

We have revised that section to read:

> *"We simulated ten phenotypes that correlated with gene expression in the observed cell type data, including testing samples, using principal component analysis (PCA). First we conducted PCA of the fully observed gene expression data in the target cell type. From each of these we derived a continuous and a binary phenotype. The continuous phenotypes were defined as the PC value plus a standard normal error. The binary phenotypes were by dichotomising each PC with values >0 designated as 1 for pseudo-cases; otherwise, 0 for pseudo-controls. For each simulated phenotype, DGE analysis was carried out twice in parallel under the limma framework [39]:*
>
> 1. *in all observed data (test + training)*
> 2. *In observed data from the training samples + imputed data from the test samples which had observed data for comparison*
>
> *We treated (1) as the ground truth, and (2) as the likely analysis adopted in any real world study. The expression matrix in (1) and (2) were used in the same limma DGE analysis. We utilised log2 transformed expression data and a predefined design matrix that incorporated simulated phenotypes (with and without sex for CLUSTER data) or treatment conditions (with and without batch for the simulation data) in the limma::lmFit function to fit the linear model, followed by empirical Bayes moderation of the standard errors using the limma::eBayes function. A false discovery rate (FDR) of 0.05 was set as the significance level for true signals in the observed data. We varied FDRs in the imputed data from 0 to 1 by steps of 0.05. Receiver operating characteristic analysis was carried out by combination of cell type, method and scenario using the pROC package "*

(i) I suggest thinking about using a different word than simulation, since, unless I misunderstand, the authors are assigning samples to phenotypes, but using the same expression. This confused me, as a simulation to me implies that counts are being simulated as well.

We have been careful to go through the manuscript and ensure we talk about "simulated phenotype" rather than "simulation" which, we agree, could suggest a complete simulated dataset.

(ii) What was the reasoning behind PC_i > 0 for stratification? Such that one gets phenotypes with continually weaker signals that are balanced in sample size between the two phenotypes?

Yes, exactly. We have now also analysed PC_i directly, to examine the performance with a quantitative phenotype, which is, after all, our motivating example. This shows similar patterns of performance.

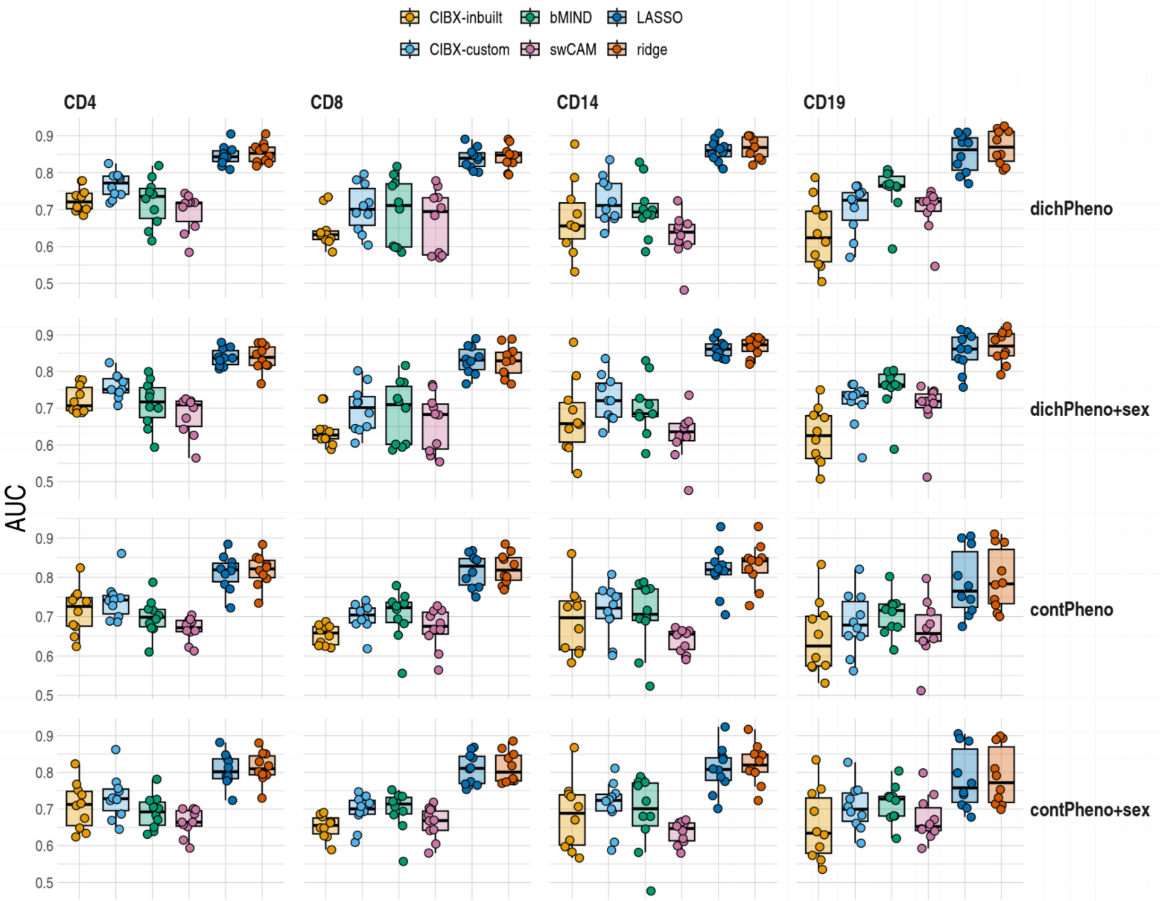

Fig 4. Differential gene expression (DGE) recovery based on CLUSTER data. Area under curve (AUC) distributions by approach and cell type (columns) for each scenario (rows). Scenarios differed in simulated dichotomous (dichPheno)/ continuous (contPheno) phenotypes, with/without sex as a covariate in the DGE models. dichPheno: dichotomous phenotype; dichPheno+sex: simulated dichotomous phenotype and sex; contPheno: continuous phenotype; contPheno+sex: continuous phenotype and sex. Each point is a simulated phenotype, and there are ten simulated phenotypes. For each simulated phenotype, the receiver operating characteristic curve and AUC were estimated by FDR fixed at 0.05 in the observed data and varied FDRs from 0 to 1 by 0.05 in the imputed data. Box plots showed the AUC distributions, with horizontal lines from the bottom to the top for 25 %, 50 % and 75 % quantiles, respectively. CIBX-inbuilt: CIBERSORTx with the inbuilt signature matrix; CIBX-custom: CIBERSORTx with a custom signature matrix; bMIND: bMIND with flow fractions; swCAM: swCAM with flow fractions; LASSO/ridge: regularised multi-response Gaussian models.

(iii) Does it make sense to include the training samples in the ground truth? This shouldn't change the methods ranking since it applies to all methods equally, but I would prefer the authors perform the data only on test samples to prevent leakage.

We included the training samples because we imagine this is how a real analysis would work, people would not discard about 50% of the available data. There are only 52-71 test samples, and so there is limited power for any method to perform well. As shown below, this in turn limits our ability to discriminate between method performance.

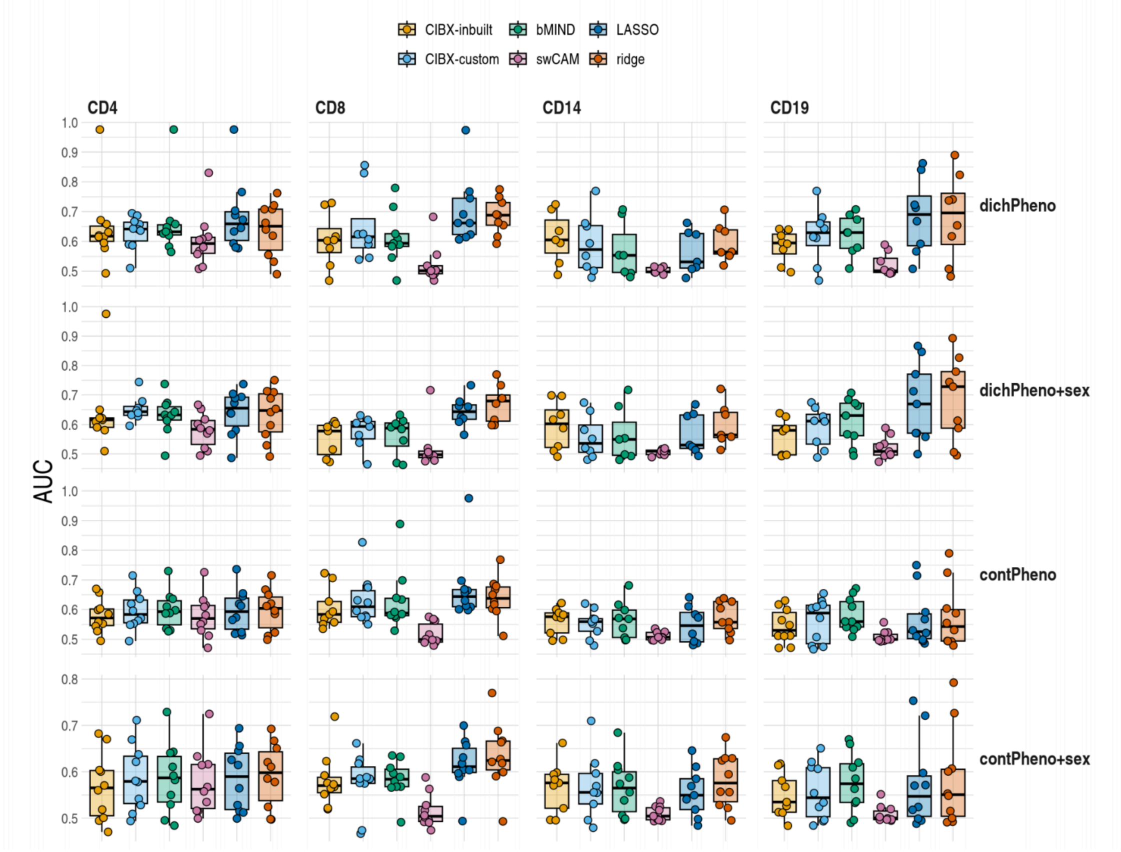

Differential gene expression (DGE) recovery. Area under curve (AUC) distributions by approach and cell type (columns) for each scenario (rows). Scenarios differed in simulated dichotomous (dichPheno)/ continuous (contPheno) phenotypes, with/without sex as a covariate in the DGE models. dichPheno: dichotomous phenotype; dichPheno+sex: simulated dichotomous phenotype and sex; contPheno: continuous phenotype; contPheno+sex: continuous phenotype and sex. **DGE analysis was carried out in test samples.** Each point is a simulated phenotype, and there are ten simulated phenotypes. For each simulated phenotype, the receiver operating characteristic curve and AUC were estimated by FDR fixed at 0.05 in the observed data and varied FDRs from 0 to 1 by 0.05 in the imputed data. Box plots showed the AUC distributions, with horizontal lines from the bottom to the top for 25 %, 50 % and 75 % quantiles, respectively. CIBX-inbuilt: CIBERSORTx with the inbuilt signature matrix; CIBX-custom: CIBERSORTx with a custom signature matrix; bMIND: bMIND with flow fractions; swCAM: swCAM with flow fractions; LASSO/ridge: regularised multi-response Gaussian models.

However, we have more power in the new pseudobulk analysis because there the association we are testing is quite a strong effect, and here we do see stronger performance of lasso and ridge.

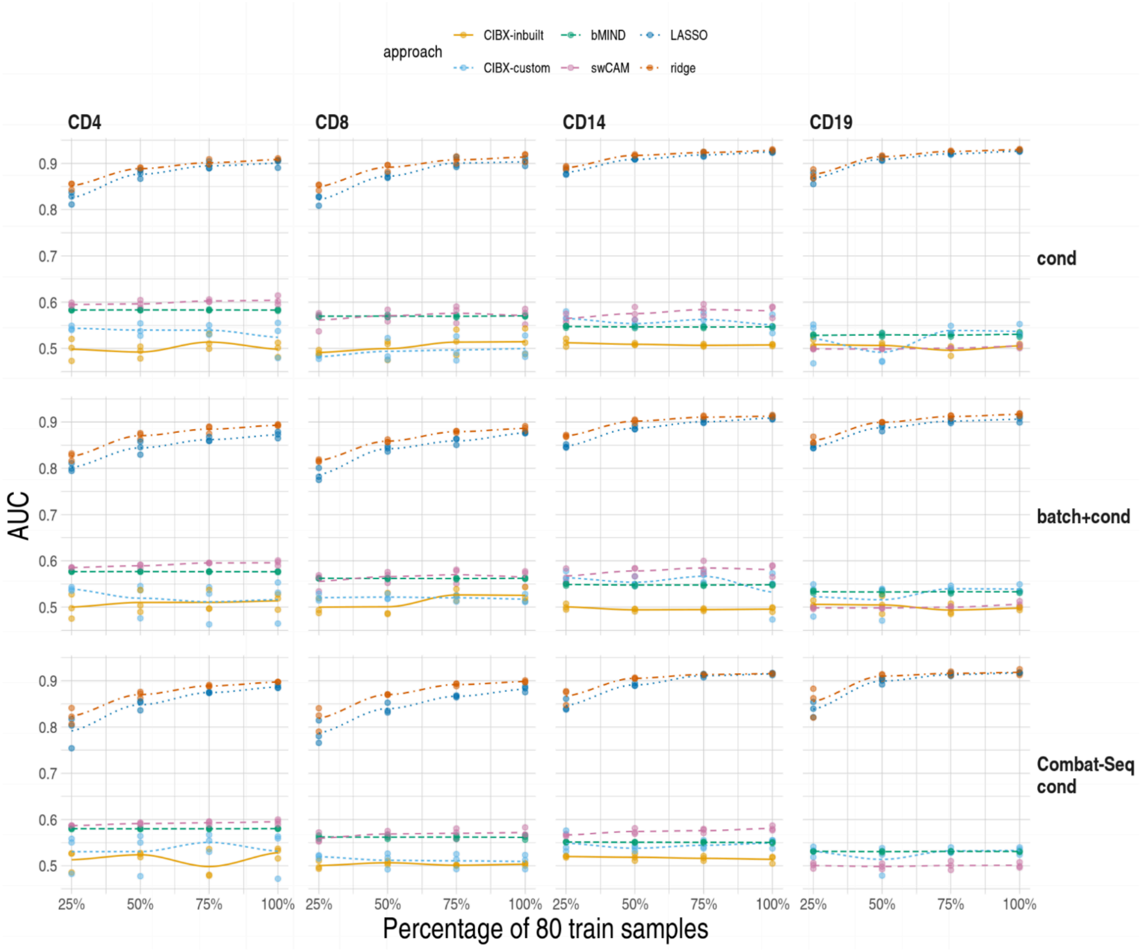

Differential gene expression (DGE) recovery based on pseudobulk data. We varied training sample size from 20 (25%) to 80 (100%) (x-axis) and quantified area under curve (AUC) for DGE recovery (y-axis) by cell type (columns) for each scenario (rows). cond: raw aggregated read counts were used for DGE analysis of in vitro stimulation with C. albicans after 3 hours (3hCA) vs untreated (UT). batch+cond: same as cond, and with batch (V2 & V3 chemistry) as a covariate in the 3hCA vs UT DGE model. Combat-seq cond: Combat-seq batch-corrected read counts were used in DGE analysis of 3hCA vs UT. **DGE analysis was performed in testing samples.** Each point is the result of one analysis, and three replicates were conducted for each training sample size. For each pseudobulk data, the receiver operating characteristic curve and AUC were estimated by FDR fixed at 0.05 in the observed DGE results and varied FDRs from 0 to 1 by 0.05 in the DGE results using imputed expression. Local polynomial regression fitting (loess) were plotted for each approach. Note that CIBX-inbuilt is not shown for CD19/Combat-Seq cond. This is because it was able to impute $<$ 60 genes, compared to $\sim$ 1,000 for CIBX-custom and $>$ 11,000 for other methods, so estimates of AUC are very noisy. CIBX-inbuilt: CIBERSORTx with the inbuilt signature matrix; CIBX-custom: CIBERSORTx with a custom signature matrix based on pure cell expression in the training samples; bMIND: bMIND with true fractions; swCAM: swCAM with true fractions; LASSO/ridge: regularised multi-response Gaussian models.

(iv) "The expression matrix used in (1) and (2) were the same in the limma DGE analysis." Can the authors clarify this sentence? Doesn't 2) use imputed data for at least some samples while 1) should only have observed data?

Imputed data were used in (2) for test samples and we simulated phenotypes based on PCA of (1). Test samples without cell type expression data can not be assigned simulated phenotypes, so they were excluded from (1) for PCA analysis and (2) for DGE analysis.

(v) To clarify: Was the DGE performed per cell type, or not? My understanding was that it had been, but then the ROC sentence sounds like only the ROC curves were calculated per cell type.

We have revised this sentence for clarity. Indeed, the ROC analysis was conducted separately for each cell type.

> *"Receiver operating characteristic analysis was carried out by combination of cell type, method and scenario using the pROC package"*

- The terminology used for RNA-seq is slightly inconsistent at times (RNA-seq vs RNAseq).

We have replaced all "RNAseq" by "RNA-seq".

- I find it fairly unfamiliar to see Ridge written in all capitals. For the Lasso, this is common as it is an acronym, but I have rarely seen it for Ridge.

This is correct. We have replaced RIDGE by ridge throughout.

- I am curious why the authors chose to correct for batch-effects. I realize that the Combat-Seq paper claims that its outputs are compatible with the count nature of common DGE methods, but I think it's still not obvious that this works (significantly) better than including batch as a covariate (especially if there is comparably little batch effect). In particular, Supplementary Figure 7 before correction looks quite fine to me even pre-batch correction.

We did this to match a common pipeline in RNA-seq differential expression which included Combat-Seq correction. Note that in S12 Fig (previously Supplementary Figure 7) batch is indicated by shape, and we can clearly distinguish batches 1 and 2 (circles and squares) from 3 and 4 (triangles and diamonds) by PC4. However, we don't believe including or excluding this step would materially affect our conclusions about the different methods compared as they all take the same input data.

- In some figures, CiberSortX is just indicated by "inbuilt" and "custom". Please add CiberSortX to this somehow as "inbuilt" and "custom" are not immediately obvious to be CiberSortX at first glance (I realize this is in the caption, but it makes scanning the figure significantly more confusing the first time reading it).

We have added the “CIBX-” prefix, consistent with Fig 2, to “inbuilt” and “custom” in the figures.

- I understand that the train/test split was stratified by batch (see e.g., Supplementary Figure 1). Have the authors also looked at the distributions of JIA and gender in the train/test and perhaps also stratified their results by either of these to make sure that no effect goes overlooked due to these? Or were these included as covariates in the DGE (in which case this was not clear/I didn't see this in the text explicitly).

The split was not stratified by batch. Instead we checked that despite a random split into test and train, batch did not differ between the two. We did not perform a similar check with sex or disease status, but as the phenotype was simulated conditional only on expression, it should not vary with sex or disease status.

- Can the authors comment on the fact that they only performed one split (i.e., no cross-validation)? I think it's fine since the datasets are very large for this type of data, but it could still be a good sanity check (as a Supplementary Figure at least) to make sure that the results are not too dependent on the specific split chosen.

We only performed one split for each simulated phenotype for computational and practical reasons. Instead, we present results over multiple simulated phenotypes, with phenotypes independent of split, which should reassure that no single unrepresentative relationship between phenotype and split is dominating. The practical reason is that we did not have data on all cell types on all subjects. Therefore some subset of samples would always need to be in the test set, and multiple train/test splits would not be independent as might be expected by readers if we used multiple splits. We have also now included additional data synthesised from single cell data, as described above, which we hope offers further reassurance.

- It would be useful to flesh out the methods section for "LASSO/RIDGE training and prediction". In particular, it may not be clear to all readers that the only way that a multitask Lasso/Ridge differs from independent (per task, i.e., per gene here) Ridge/Lassos is via the (grouped) regularization term (e.g., forcing all tasks to have the same non-zero coefficients for the Lasso), so it would be useful to write down the objective function to clarify this, I think.

We have now explained multitask penalized regressions more clearly in the methods:

> *"Standard penalised regression approaches aim to predict a single variable given observations of multiple predictors. While ordinary least squares aims to identify* β *that minimises* $\sum_{i}^{N}(y_i - \beta_0 - x_i\beta)^2$ *for predictors* x_i *and outcomes* y_i*, penalised regression approaches do this subject to constraints:* $\sum_j |\beta_j| \leq \alpha$ *(LASSO) or* $\sum_j \beta_j^2 \leq \alpha$ *(ridge). We could train a separate LASSO model using all genes in the mixed cell data to predict each gene in a sorted cell in turn. However, given the correlations between expressions of different genes, we hypothesised that multi-response models may be more accurate given they allow sharing of information between the genes to be predicted. In a multi-response model, all genes in the mixed cell data are still used but now prediction is made for a set of genes. Information is shared a coefficients for any predictor are either zero for all target genes or non-zero for all target genes. However, note that non-zero coefficients have distinct values for each target gene. Thus multi-response models are appropriate to predict subsets of correlated genes."*

- Please also give additional details on which DGE workflow in limma was exactly carried out.

We have included the following sentence detailing the limma DGE workflow we used.

> *"We utilised log2 transformed expression data and a predefined design matrix that incorporated simulated phenotypes (with and without sex for CLUSTER data) or treatment conditions (with and without batch for the simulation data) in the limma::lmFit function to fit the linear model, followed by empirical Bayes moderation of the standard errors using the limma::eBayes function."*

Have the authors made all data and (if applicable) computational code underlying the findings in their manuscript fully available?

The PLOS Data policy requires authors to make all data and code underlying the findings described in their manuscript fully available without restriction, with rare exception (please refer to the Data Availability Statement in the manuscript PDF file). The data and code should be provided as part of the manuscript or its supporting information, or deposited to a public repository. For example, in addition to summary statistics, the data points behind means, medians and variance measures should be available. If there are restrictions on publicly sharing data or code —e.g. participant privacy or use of data from a third party—those must be specified.

Reviewer #1: Yes

Reviewer #2: Yes

Reviewer #3: Yes

PLOS authors have the option to publish the peer review history of their article (what does this mean?). If published, this will include your full peer review and any attached files.

If you choose “no”, your identity will remain anonymous but your review may still be made public.

Do you want your identity to be public for this peer review? For information about this choice, including consent withdrawal, please see our Privacy Policy.

Reviewer #1: No

Reviewer #2: No

Reviewer #3: No

Figure Files:

While revising your submission, please upload your figure files to the Preflight Analysis and Conversion Engine (PACE) digital diagnostic tool, https://pacev2.apexcovantage.com. PACE helps ensure that figures meet PLOS requirements. To use PACE, you must first register as a user. Then, login and navigate to the UPLOAD tab, where you will find detailed instructions on how to use the tool. If you encounter any issues or have any questions when using PACE, please email us at figures@plos.org.

Data Requirements:

Please note that, as a condition of publication, PLOS' data policy requires that you make available all data used to draw the conclusions outlined in your manuscript. Data must be deposited in an appropriate repository, included within the body of the manuscript, or uploaded as supporting information. This includes all numerical values that were used to generate graphs, histograms etc.. For an example in PLOS Biology see here:

http://www.plosbiology.org/article/info%3Adoi%2F10.1371%2Fjournal.pbio.1001908#s5
.

Reproducibility:

To enhance the reproducibility of your results, we recommend that you deposit your laboratory protocols in protocols.io, where a protocol can be assigned its own identifier (DOI) such that it can be cited independently in the future. Additionally, PLOS ONE offers an option to publish peer-reviewed clinical study protocols. Read more information on sharing protocols at
https://plos.org/protocols?utm_medium=editorial-email&utm_source=authorletters&utm_campaign=protocols

Dear Dr Wallace

I hope this email finds you well.

Since we sent the decision, we have received an addition to the comments by the Reviewer 3.

Please see this information here:

****[1] Jaakkola, Maria K., and Laura L. Elo. "Estimating cell type-specific differential expression using deconvolution." Briefings in Bioinformatics 23.1 (2022): bbab433.

[2] Meng, Guanqun, et al. "A comprehensive assessment of cell type-specific differential expression methods in bulk data." Briefings in Bioinformatics 24.1 (2023): bbac516.

[3] Tang, Daiwei, Seyoung Park, and Hongyu Zhao. "SCADIE: simultaneous estimation of cell type proportions and cell type-specific gene expressions using SCAD-based iterative estimating procedure." Genome Biology 23.1 (2022): 129.*****

If you have any further queries or concerns, please don't hesitate to get back in touch.

Kind Regards,

Olena Szabo

PLOS Computational Biology
