# Supplementary material for: Penalised regression improves imputation of cell-type specific expression using RNA-seq data from mixed cell populations compared to domain-specific methods
Source: PLoS Comput Biol. 2025 Mar 7;21(3):e1012859. doi: 10.1371/journal.pcbi.1012859 (PMC11957391; doi:10.1371/journal.pcbi.1012859)
Supplement: Attachment — Submitted filename: PCOMPBIOL-R1-Rebuttal.pdf [file pcbi.1012859.s020.pdf]

Dear Dr. Wallace,

Thank you for submitting your manuscript to PLOS Computational Biology. After careful consideration, we feel that it has merit but does not fully meet PLOS Computational Biology's publication criteria as it currently stands. Therefore, we invite you to submit a revised version of the manuscript that addresses the points raised during the review process.

I especially agree with the need to correct for small mistakes of writing in the newly added text. You may treat all other points of reviewer 2 as optional, although I ask you to consider them carefully and provide a detailed responses.

Please submit your revised manuscript within 30 days **Feb 15 2025 11:59PM**. If you will need more time than this to complete your revisions, please reply to this message or contact the journal office at ploscompbiol@plos.org. When you're ready to submit your revision, log on to https://www.editorialmanager.com/pcompbiol/ and select the 'Submissions Needing Revision' folder to locate your manuscript file.

Please include the following items when submitting your revised manuscript:

* A rebuttal letter that responds to each point raised by the editor and reviewer(s). You should upload this letter as a separate file labeled 'Response to Reviewers'. This file does not need to include responses to formatting updates and technical items listed in the 'Journal Requirements' section below.
* A marked-up copy of your manuscript that highlights changes made to the original version. You should upload this as a separate file labeled 'Revised Manuscript with Track Changes'.
* An unmarked version of your revised paper without tracked changes. You should upload this as a separate file labeled 'Manuscript'.

If you would like to make changes to your financial disclosure, competing interests statement, or data availability statement, please make these updates within the submission form at the time of resubmission. Guidelines for resubmitting your figure files are available below the reviewer comments at the end of this letter.

We look forward to receiving your revised manuscript.

Kind regards,

Marc Robinson-Rechavi

Academic Editor
PLOS Computational Biology

Ilya Ioshikhes
Section Editor
PLOS Computational Biology

Journal Requirements:

Please ensure that the funders and grant numbers match between the Financial Disclosure field and the Funding Information tab in your submission form. Note that the funders must be provided in the same order in both places as well.

Reviewers' comments:

Reviewer's Responses to Questions

Comments to the Authors:
Please note here if the review is uploaded as an attachment.

Reviewer #1: The authors have addressed all my concerns.

Reviewer #2: I appreciate the revisited version of the manuscript, especially the effort to make the mathematical notation more clear has made the paper easier to follow.
However, I still think that a few points need to be addressed further.
1. The definition of the LASSO and RIDGE regression needs to be further clarified. In fact, while I the equation with the predictor and observed variable is enough to summarise the regression, since the authors report in the manuscript that these general ML models have never been applied in this context and they are independent on the deconvolution equation, I believe it is necessary to explain what the predictor variables and their coefficients beta correspond to.
I might be missing a point but to me this is not clear. For example, are all x-s in the regression binary variable values corresponding to each gene, while the betas correspond to their expression?

> The variables are the expression values, with one variable corresponding to one gene in the mixed cell expression data. The coefficients relate the expression of each gene in the mixed cell expression data to the expression of a target gene in the

sorted cell expression data. We have clarified this in the section “LASSO/ridge training and prediction” in the Methods:

“Ordinary least squares aims to identify β that minimises $\sum_{i}^{N}(y_i - \beta_0 - \sum_{j}^{p} x_{ij}\beta_j)^2$ for predictors x_j and outcomes y measured in subjects $i = 1,..., N$. In our application, y is the expression of a target gene in a specific cell type, x_j is the expression of gene j in PBMCs, β_0 is an intercept term and β_j is the coefficient to predict y from x_j. Penalised regression approaches minimise this quantity subject to constraints: $\sum_j |\beta_j| \leq \alpha$ (LASSO) or $\sum_j \beta_j^2 \leq \alpha$ (ridge).”

2. Given that the procedure to apply LASSO/ridge regression involves different steps (e.g., the hierarchical clustering of genes), I think that a graphical representation of the method would increase the clarity of the manuscript.

We have generated a new figure (Fig 1) to illustrate steps we use in multi-response LASSO/ridge models on the training set to predict cell-type expression for test samples

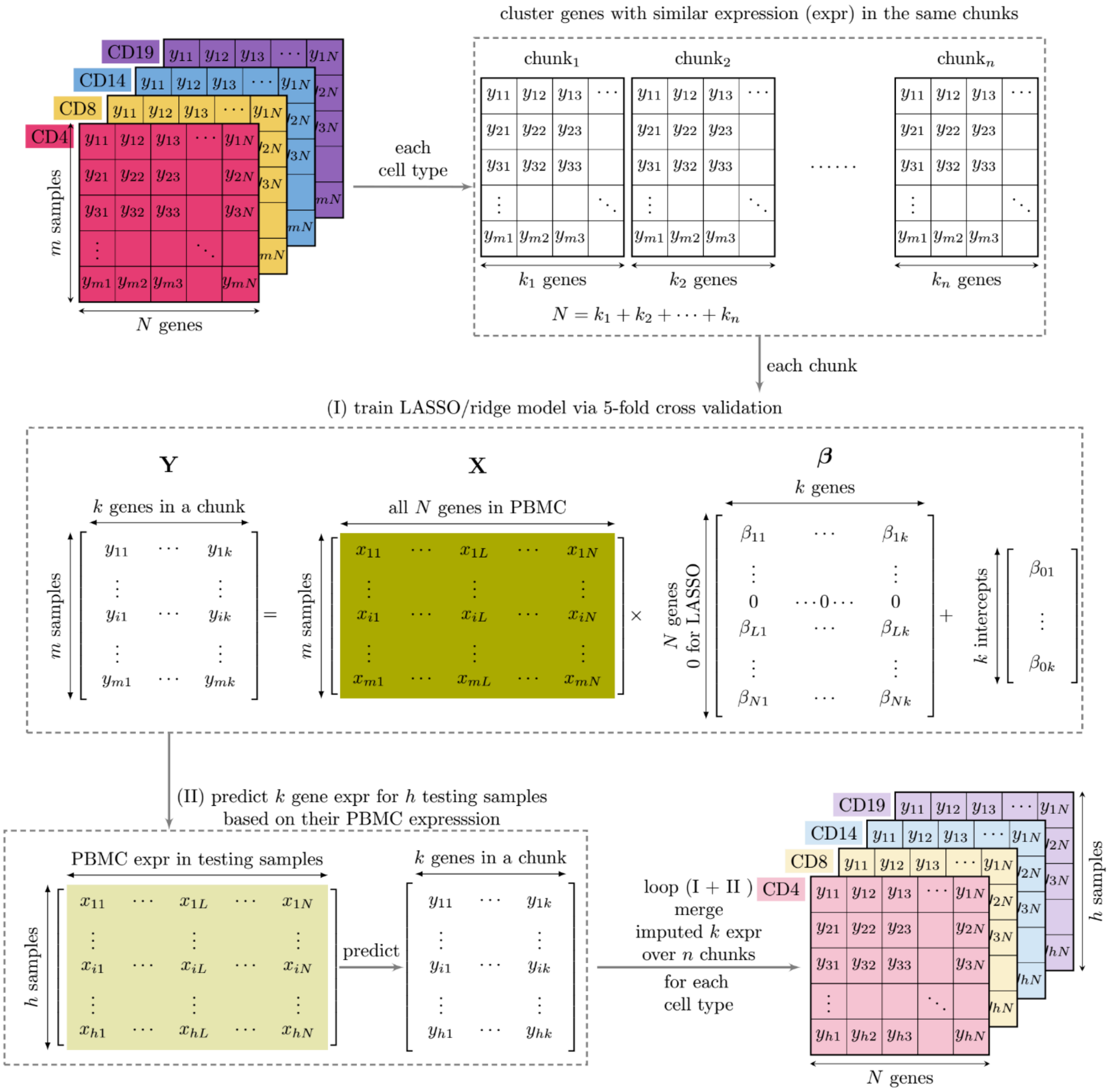

Fig 1. Multi-response LASSO/ridge models for predicting sample-level cell-type expression. We utilised gene expression data from pure cell types (such as CD4, CD8, CD14, and CD19) and a mixed cell type (such as PBMC), all obtained from the same subjects as our training data. For each cell type, we clustered genes with similar expression into chunks. For each chunk, we learned the expression associations between cell-type-specific target genes and predictor genes in PBMC using a multi-response LASSO/ridge model with five-fold cross-validation. The multi-response model includes a group penalty so that regression coefficients $\boldsymbol{\beta}$ for any given predictor may be shrunk to zero for all target genes. The non-zero $\boldsymbol{\beta}$ take different values corresponding to each target gene. This multi-response LASSO/ridge model was then employed to predict the expression of cell-type-specific target genes in testing samples based on their PBMC expression. The learning and prediction steps were repeated for all gene chunks for each cell type, and the predicted target gene expression was assembled on a per-cell type basis.

3. Regarding the new test on eQTL data, what data is used to compute the AUROC measure? More specifically, when 75% of the samples are used for training, is the remainder 25% used for testing? Or is the AUROC computed on the same 75% points used for training?

> Our AUC are always calculated on held out testing data. In the simulations, we say in the Methods
>
> *“For each of the six simulation datasets, we designated the first half of samples as the training set and the rest as the testing set. We then varied the sample sizes of training samples, from 25% (N=20) to 100% (N=80) by 25%, while keeping the same testing samples.”*
>
> The AUROC is computed on the held out test data. We have clarified this in the methods
>
> *“All cells used to generate data for any sample were either from cells stimulated with C. albicans or untreated. We evaluated every trained model’s ability to predict the outcome of in vitro stimulation in the held out test data, quantifying this by AUC”*.
>
> We have also clarified the legends of original Fig 4 and 5 (now Fig 5 and 6) to say that the AUC is estimated in held out test data.

Minor comments:
- There are few typos throughout the text. For example on page 21
Information is shared a coefficients for any predictor are either zero for all target genes or non-zero for all target genes
- On page 6: "We and compare the these domain-specific to general machine learning methods"

> We apologise for these typos which arose through using track changes and which have now been corrected. We have carefully read through the manuscript to identify any further examples.

Have the authors made all data and (if applicable) computational code underlying the findings in their manuscript fully available?
The PLOS Data policy requires authors to make all data and code underlying the findings described in their manuscript fully available without restriction, with rare exception (please

refer to the Data Availability Statement in the manuscript PDF file). The data and code should be provided as part of the manuscript or its supporting information, or deposited to a public repository. For example, in addition to summary statistics, the data points behind means, medians and variance measures should be available. If there are restrictions on publicly sharing data or code —e.g. participant privacy or use of data from a third party—those must be specified.

Reviewer #1: Yes

Reviewer #2: Yes

PLOS authors have the option to publish the peer review history of their article (what does this mean?). If published, this will include your full peer review and any attached files.

If you choose "no", your identity will remain anonymous but your review may still be made public.

Do you want your identity to be public for this peer review? For information about this choice, including consent withdrawal, please see our Privacy Policy.

Reviewer #1: No

Reviewer #2: No

[NOTE: If reviewer comments were submitted as an attachment file, they will be attached to this email and accessible via the submission site. Please log into your account, locate the manuscript record, and check for the action link "View Attachments". If this link does not appear, there are no attachment files.]

Figure resubmission:

While revising your submission, please upload your figure files to the Preflight Analysis and Conversion Engine (PACE) digital diagnostic tool, https://pacev2.apexcovantage.com/. PACE helps ensure that figures meet PLOS requirements. To use PACE, you must first register as a user. Registration is free. Then, login and navigate to the UPLOAD tab, where you will find detailed instructions on how to use the tool. If you encounter any issues or have any questions when using PACE, please email PLOS at figures@plos.org. Please note

that Supporting Information files do not need this step. If there are other versions of figure files still present in your submission file inventory at resubmission, please replace them with the PACE-processed versions.

Reproducibility:

To enhance the reproducibility of your results, we recommend that authors of applicable studies deposit laboratory protocols in protocols.io, where a protocol can be assigned its own identifier (DOI) such that it can be cited independently in the future. Additionally, PLOS ONE offers an option to publish peer-reviewed clinical study protocols. Read more information on sharing protocols at https://plos.org/protocols?utm_medium=editorial-email&utm_source=authorletters&utm_campaign=protocols
